# Pregnancy‐related mortality up to 1 year postpartum in sub‐Saharan Africa: an analysis of verbal autopsy data from six countries

**DOI:** 10.1111/1471-0528.17606

**Published:** 2023-07-19

**Authors:** Ursula Gazeley, Georges Reniers, Julio E. Romero‐Prieto, Clara Calvert, Momodou Jasseh, Kobus Herbst, Sammy Khagayi, David Obor, Daniel Kwaro, Albert Dube, Merga Dheresa, Chodziwadziwa W. Kabudula, Kathleen Kahn, Mark Urassa, Amek Nyaguara, Marleen Temmerman, Laura A. Magee, Peter von Dadelszen, Veronique Filippi

**Affiliations:** ^1^ Department of Infectious Disease Epidemiology London School of Hygiene and Tropical Medicine London UK; ^2^ Department of Population Health London School of Hygiene and Tropical Medicine London UK; ^3^ Usher Institute, University of Edinburgh Edinburgh UK; ^4^ Medical Research Council Unit The Gambia at LSHTM Serekunda The Gambia; ^5^ Africa Health Research Institute Durban South Africa; ^6^ DSI‐MRC South African Population Research Infrastructure Network (SAPRIN) Durban South Africa; ^7^ Kenya Medical Research Institute – Centre for Global Health Research Kisumu Kenya; ^8^ Malawi Epidemiology and Intervention Research Institute Karonga Malawi; ^9^ School of Nursing and Midwifery, College of Health and Medical Sciences Haramaya University Harar Ethiopia; ^10^ MRC/Wits Rural Public Health and Health Transitions Research Unit (Agincourt), School of Public Health, Faculty of Health Sciences University of the Witwatersrand Johannesburg South Africa; ^11^ Department of Epidemiology and Global Health Umeå University Umeå Sweden; ^12^ The Tazama Project, National Institute for Medical Research Mwanza Tanzania; ^13^ KEMRI‐Wellcome Trust Kilifi Kenya; ^14^ Centre of Excellence in Women and Children's Health Aga Khan University Nairobi Kenya; ^15^ Department of Women and Children's Health, School of Life Course and Population Sciences, Faculty of Life Science and Medicine King's College London London UK; ^16^ Institute of Women and Children's Health, King's College London London UK

**Keywords:** causes of death, maternal health, pregnancy‐related mortality, verbal autopsy

## Abstract

**Objective:**

To compare the causes of death for women who died during pregnancy and within the first 42 days postpartum with those of women who died between >42 days and within 1 year postpartum.

**Design:**

Open population cohort (Health and Demographic Surveillance Systems).

**Setting:**

Ten Health and Demographic Surveillance Systems (HDSS) in The Gambia, Kenya, Malawi, Tanzania, Ethiopia and South Africa.

**Population:**

2114 deaths which occurred within 1 year of the end of pregnancy where a verbal autopsy interview was conducted from 2000 to 2019.

**Methods:**

InterVA5 and InSilicoVA verbal autopsy algorithms were used to attribute the most likely underlying cause of death, which were grouped according to adapted International Classification of Diseases‐Maternal Mortality categories. Multinomial regression was used to compare differences in causes of deaths within 42 days versus 43–365 days postpartum adjusting for HDSS and time period (2000–2009 and 2010–2019).

**Main outcome measures:**

Cause of death and the verbal autopsy Circumstances of Mortality Categories (COMCATs).

**Results:**

Of 2114 deaths, 1212 deaths occurred within 42 days postpartum and 902 between 43 and 365 days postpartum. Compared with deaths within 42 days, deaths from HIV and TB, other infectious diseases, and non‐communicable diseases constituted a significantly larger proportion of late pregnancy‐related deaths beyond 42 days postpartum, and health system failures were important in the circumstances of those deaths. The contribution of HIV and TB to deaths beyond 42 days postpartum was greatest in Southern Africa. The causes of pregnancy‐related mortality within and beyond 42 days postpartum did not change significantly between 2000–2009 and 2010–2019.

**Conclusions:**

Cause of death data from the extended postpartum period are critical to inform prevention. The dominance of HIV and TB, other infectious and non‐communicable diseases to (late) pregnancy‐related mortality highlights the need for better integration of non‐obstetric care with ante‐, intra‐ and postpartum care in high‐burden settings.

## 1 INTRODUCTION

Globally, remarkably little is known about the causes of death (COD) for women who die beyond the standard 42‐day postpartum period,^1^ despite the need for this evidence to inform policy and programming to prevent these deaths. This evidence gap is particularly apparent in sub‐Saharan Africa. Of five relevant studies on the causes of deaths in the extended postpartum, two did not disaggregate deaths within 42 days postpartum from those that occurred after 42 days;^2^, ^3^ two had small samples and are now outdated;^4^, ^5^ and one study in South Africa identified HIV and TB as the primary causes of deaths beyond 42 days postpartum.^6^

The reasons for the lack of data on deaths beyond 42 days are twofold. First, although late maternal deaths occurring beyond 42 days but within 1 year of the end of pregnancy are defined (‘any cause related to or aggravated by the pregnancy or its management but not from unintentional or incidental causes’^7^) only maternal deaths within 42 days are included in the numerator of the Maternal Mortality Ratio (MMR) – the primary indicator used to monitor trends in maternal survival between countries and over time. For pregnancy‐related deaths (‘a female death occurring within 42 days of termination of pregnancy, irrespective of cause’^7^), there is no corollary definition of what could be called ‘late pregnancy‐related mortality’ for deaths between 43 and 365 days postpartum, despite recent evidence that the risk of pregnancy‐related mortality remains elevated until 4 months after delivery.^8^ Many countries, therefore, either do not monitor or do not report estimates of mortality levels and causes beyond 42 days postpartum.

Secondly, cause of death information and analyses in sub‐Saharan Africa frequently falls short of international standards.^9^ Civil Registration and Vital Statistics systems and medical certification of the cause of death are often incomplete,^9^, ^10^ and though many African countries have Maternal and Perinatal Death Surveillance and Response (MPDSR) with a national policy for maternal deaths notification,^11^ MPDSR systems rarely review deaths beyond 42 days postpartum. Deaths that occur outside of the labour or postnatal ward or cases that were referred to higher‐level facilities may also be less likely to be captured and reviewed through MPDSR.^12^ Finally, MPDSRs are primarily facility‐based; community deaths during pregnancy and the extended postpartum period may be missed when community reporting mechanisms are weak.^12^ These concerns may be particularly salient for deaths occurring after 42 days postpartum.

For these reasons, population‐based HDSS and verbal autopsy (VA) data are essential to estimate the causes of pregnancy‐related deaths up to 1 year postpartum in data‐scarce contexts. Our objective was to compare the causes of pregnancy‐related deaths occurring during pregnancy and within 42 days postpartum with ‘late’ pregnancy‐related deaths occurring 43 days to 1 year postpartum to provide much‐needed evidence on the causes of death in the extended postpartum in sub‐Saharan Africa.

## 2 METHODS

### 2.1 Data

This study pooled longitudinal, prospective data from 10 Health and Demographic Surveillance Systems (HDSS) across six countries in sub‐Saharan Africa: Basse and Farafenni (The Gambia), Nairobi, Kisumu and Kilifi (Kenya), Agincourt and uMkhanyakude (South Africa), Karonga (Malawi), Kersa (Ethiopia), and Kisesa (Tanzania). HDSS are open population cohorts and collect data on births and deaths that occur within a small geographical area quarterly to biannually. Each HDSS had their own informed consent procedure (either written or verbal), which in most sites was at the household level.^13^ Access to data for Agincourt, Basse, Farafenni, Kersa, Kisesa and Kisumu was arranged through data‐sharing agreements with the London School of Hygiene and Tropical Medicine (LSHTM). Access to data for Karonga, Kilifi, Nairobi and uMkhanyakude was granted through the sites’ online data repositories. The LSHTM ethics committee approved this study.

### 2.2 Study population

Deaths that occurred in the 20‐year period from 2000 to 2019 (inclusive) were included. Deaths pre‐2000 were excluded because only a few sites were operational; deaths occurring between 2020 and 2022 were excluded to limit the effect of COVID‐19 misclassification, with COVID‐19 only introduced to the WHO verbal autopsy tool in 2022.^14^

We identified all deaths of women aged 10–54 years that occurred up to 1 year postpartum in two ways. First, we identified postpartum deaths for whom the date of the end of pregnancy was available in the HDSS delivery file and for whom a VA interview was conducted. In all 10 sites, this included pregnancies ending in a live birth or stillbirth after 28 weeks’ gestation. In Basse, Farafenni, Kilifi and Nairobi, pregnancy terminations before 28 weeks’ gestation (miscarriage or abortion) were also included. We then identified which of these women died within 1 year postpartum. Secondly, we identified all additional deaths where the proxy respondent reported in the VA interview that the woman was either currently pregnant or in labour at the time of death, or died within 42 days of pregnancy termination, but for whom no delivery was recorded in the HDSS delivery file.

### 2.3 Procedures

Deaths were grouped as occurring during pregnancy and within the standard 42‐day postpartum period or between 43 days and 1 year postpartum. Where a discrepancy existed between the timing of death in the VA data and HDSS delivery data (*n* = 41), priority was given to the VA data if it was possible that a repeat pregnancy had occurred that had not yet been recorded in the HDSS delivery file. Maternal age at death was grouped by 5‐year intervals from 10–14 to 50–54 years.

For each death, we estimated the single most likely underlying COD using the InterVA5 algorithm. We grouped deaths according to the four types and nine adapted International Classification of Diseases‐Maternal Mortality (ICD‐MM) categories^15^, ^16^ as follows:

*Obstetric* (1. Pregnancy with abortive outcome, 2. Hypertensive disorders, 3. Obstetric haemorrhage, 4. Pregnancy‐related infection, 5. Other obstetric complications, 6. Unanticipated complications of management);

*Non‐obstetric* (7a. HIV & TB, 7b. Other infectious diseases, 7c. Cardiovascular diseases, 7d. Other NCDs);

*Unspecified* (8. Undetermined);

*External* (9. Accidents & violence).

Exact replication of the ICD‐MM categories was not possible because we analysed pregnancy‐related deaths (i.e. defined only by time of death) and not maternal mortality. From VA data alone, it is not possible to differentiate which non‐obstetric pregnancy‐related deaths were indirect maternal deaths and which were coincidental; this would require a clinical COD expert reviewing a patients’ medical records to ascertain whether the underlying condition (e.g. HIV, carcinoma or cardiovascular disease) was ‘aggravated by pregnancy’ – as is required for the death to be considered maternal. This was not possible without further record linkage and data triangulation, and hence we modified the ICD‐MM categories to apply to pregnancy‐related mortality (Figure S1). *Obstetric* and *Unspecified* groups replicate the ICD‐MM categories. *Non‐obstetric* includes all non‐obstetric causes without an attribution whether the death was indirect maternal death or coincidental to the pregnancy. *External* includes deaths from accidents and violent injuries only.

Finally, for each death, we processed the Circumstances of Mortality categories (COMCATs) using InterVA5 and COMCAT version 1.0 to attribute the most likely circumstance of mortality: traditions, emergencies, recognition of serious disease, resources, health systems, inevitability and multiple.

### 2.4 Statistical analyses

Based on the classification of death type and adapted ICD‐MM category, we estimated the relative COD distribution by age group and whether the death occurred within or beyond 42 days postpartum. Multinomial logistic regression was used to calculate the predictive margins (potential‐outcome means) for deaths occurring within and after 42 days postpartum, to adjust for potential confounders – maternal age at death, time period and HDSS. As each ICD‐MM category is a competing cause of death, we estimated one enclosing multinomial model for all causes.

We tested the sensitivity of the results to the choice of VA algorithm, comparing InterVA5 with InSilicoVA. We also compared the concordance between algorithm‐ and physician‐coded VA for the underlying cause, adapted ICD‐MM categories, and type (Kisumu, Nairobi and Karonga, only), and the concordance of InterVA5 and InSilicoVA algorithms for all 10 HDSS.

## 3 RESULTS

Between 3 January 2000 and 21 December 2019, there were 2114 deaths during pregnancy and up to 1 year postpartum in the HDSS, of which 902 (42.7%) occurred beyond 42 days postpartum.

The background characteristics of these deaths are presented in Table 1. For each HDSS, the years of the earliest to last death are presented in parentheses. Except in Agincourt and uMkhanyakude, more deaths occurred during pregnancy and within 42 days than from 43 to 365 days postpartum. Almost two‐thirds of deaths from 43 to 365 days postpartum occurred between the years 2000 and 2009.

**TABLE 1:** Background characteristics.

Characteristic	Pregnancy‐related deaths	
	Deaths during pregnancy and within 42 days (inclusive) postpartum, *n* = 1212	Deaths from 43 to 365 days postpartum, *N* = 902
HDSS^a^ (earliest to last death in sample)		
Agincourt, South Africa (2000–2019)	127 (10.5%)	131 (14.5%)
Basse, The Gambia (2006–2018)	143 (11.8%)	62 (6.9%)
Farafenni, The Gambia (2000–2018)	74 (6.1%)	15 (1.7%)
Karonga, Malawi (2003–2017)	58 (4.8%)	31 (3.4%)
Kersa, Ethiopia (2008–2019)	36 (3.0%)	23 (2.5%)
Kilifi, Kenya (2008–2019)	160 (13.1%)	69 (7.7%)
Kisumu, Kenya (2003–2013)	328 (27.1%)	281 (31.1%)
Magu, Tanzania (2000–2016)	71 (5.9%)	23 (2.6%)
Nairobi, Kenya (2003–2016)	88 (7.3%)	57 (6.3%)
uMkhanyakude, South Africa (2000–2019)	127 (10.5%)	210 (23.3%)
Age at death		
10–14	1 (0.1%)	1 (0.1%)
15–19	134 (11.1%)	50 (5.6%)
20–24	304 (25.1%)	205 (22.7%)
25–29	289 (23.8%)	270 (29.9%)
30–34	247 (20.4%)	196 (21.7%)
35–39	155 (12.8%)	126 (14.0%)
40–44	69 (5.7%)	44 (4.9%)
45–49	11 (0.9%)	10 (1.1%)
50–54	2 (0.2%)	0 (0.0%)
Year of death		
2000–2009	679 (56.0%)	556 (61.6%)
2010–2019	533 (44.0%)	346 (38.4%)

^a^ Health and Demographic Surveillance System.

Table 2 shows the full results for InterVA5, with the breakdown of deaths by type, adapted ICD‐MM category and underlying cause. There were no deaths attributed to unanticipated complications of management in the data. Most other direct obstetric deaths were unspecified maternal causes; malaria and pneumonia were the leading causes of other infectious diseases; and digestive neoplasms were the leading cause of other NCDs.

**TABLE 2:** Underlying causes and ICD‐MM cause categories, InterVA5.

Adapted ICD‐MM^a^ group	Underlying cause	Deaths within 42 days	Deaths from 43 to 365 days
Obstetric causes		728	63
1. Abortive	Abortion‐related death	46	8
	Ectopic pregnancy	17	0
2. Hypertensive	Pregnancy‐induced hypertension	96	15
3. Obstetric haemorrhage	Obstetric haemorrhage	456	18
	Ruptured uterus	12	1
4. Pregnancy‐related infection	Pregnancy‐related sepsis	53	8
5. Other direct obstetric	Anaemia of pregnancy	15	2
	Intentional self‐harm	0	11
	Obstructed labour	3	0
	Other and unspecified maternal cause	30	0
6. Unanticipated complications of management	N/A	0	0
Non‐obstetric causes	422	758	
7a. HIV/tuberculosis	HIV‐related death	110	317
	Tuberculosis	61	125
7b. Other infectious diseases	Acute respiratory infection including pneumonia	36	41
	Diarrhoeal diseases	2	18
	Haemorrhagic fever (non‐dengue)	0	2
	Malaria	32	34
	Meningitis and encephalitis	13	19
	Other and unspecified infectious disease	3	11
7c. Cardiovascular diseases	Acute cardiac disease	11	9
	Other and unspecified cardiac disease	72	35
	Stroke	6	9
7d. Other NCDs^b^	Acute abdomen	11	16
	Asthma	4	3
	Breast neoplasms	0	7
	Diabetes mellitus	7	7
	Digestive neoplasms	10	30
	Epilepsy	6	6
	Liver cirrhosis	7	5
	Oral neoplasms	1	0
	Other and unspecified NCD	4	6
	Other and unspecified neoplasms	1	11
	Renal failure	2	8
	Reproductive neoplasms	14	25
	Respiratory neoplasms	7	11
	Severe malnutrition	0	2
	Sickle cell with crisis	2	1
Unspecified cause		37	43
8. Undetermined	Undetermined	37	43
External causes		25	38
9. Accidents & violence	Accidental drowning and submersion	3	3
	Accidental exposure to smoke fire & flame	0	4
	Accidental fall	0	2
	Assault	9	11
	Road traffic accident	13	18
Total		1212	902

^a^ International Classification of Diseases – Maternal Mortality.

^b^ Non‐communicable diseases.

**FIGURE 1:**
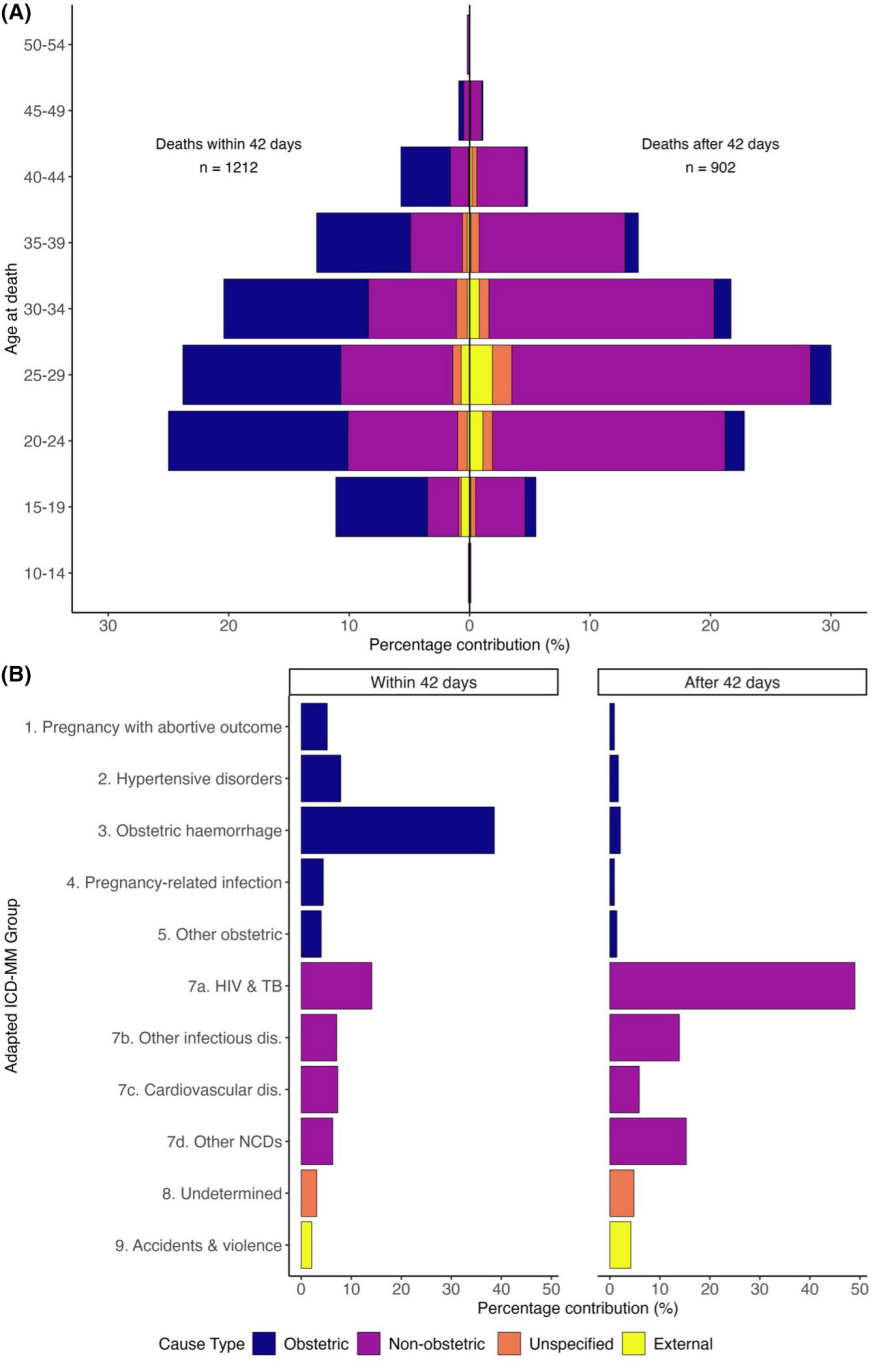
Panel A: Causes of pregnancy‐related deaths up to 1 year postpartum by timing and age;Panel B: Causes of pregnancy‐related deaths up to 1 year postpartum by timing and ICD‐MM category. There were no deaths attributed to ICD‐MM category 6. Unanticipated complications of management, so this category is not shown.

Figure 1A shows the proportion of pregnancy‐related deaths for each COD type for data from all 10 HDSS pooled together. Across all age groups, for deaths occurring during pregnancy and within 42 days postpartum, direct obstetric causes were the leading COD, whereas for deaths beyond 42 days, non‐obstetric causes were dominant. Undetermined and external causes constituted a small proportion of deaths across all age groups and each period.

Figure 1B shows the proportion of pregnancy‐related deaths for each type disaggregated by adapted ICD‐MM category for all 10 HDSS. For deaths occurring within 42 days, obstetric haemorrhage was the dominant cause, followed by HIV and TB. The proportion of deaths from hypertensive disorders was comparable to deaths from other infectious diseases and cardiovascular diseases. For late pregnancy‐related deaths occurring from 43 to 365 days postpartum, HIV and TB were the dominant causes, followed by other NCDs and other infectious diseases. All obstetric causes constituted a small proportion of the late pregnancy‐related deaths. The timing of obstetric deaths from 43 days to 1 year postpartum is shown in Figure S2.

Multinomial logistic regression confirmed that the predicted proportions for all direct obstetric causes of pregnancy‐related deaths were significantly larger for deaths occurring within (versus beyond) 42 days postpartum, adjusting for HDSS and time period (2000–2009 and 2010–2019). Maternal age at death was not significant and was dropped from the final model. After adjusting for time period and HDSS heterogeneity, HIV & TB, other infectious diseases and other NCDs were significantly more likely causes of pregnancy‐related deaths occurring beyond (versus within) 42 days postpartum. For full multinomial results see Table S1 and Figure S3.

Sensitivity analyses show that the results for InSilicoVA were broadly consistent, although within 42 days postpartum, the contribution of pregnancy‐related infection was slightly higher and that of cardiovascular disease was lower compared with InterVA5; replication of the main results can be found in Figure S4. Concordance between physician‐coded VA and algorithm‐assigned results can be found in Table S2 for the three HDSS with available physician‐coded VA data; concordance of physician‐coded VA results was low for both the underlying cause of death (ranging from 25% to 43%) and adapted ICD‐MM category (from 33% to 55%), but was higher for broad type (from 82% to 83%). Agreement between InterVA55 and InSilicoVA was high for all 10 HDSS (from 47% for underlying cause to 93% for broad cause type).

### 3.1 Causes of pregnancy‐related deaths from 2000–2009 and 2010–2019

**FIGURE 2:**
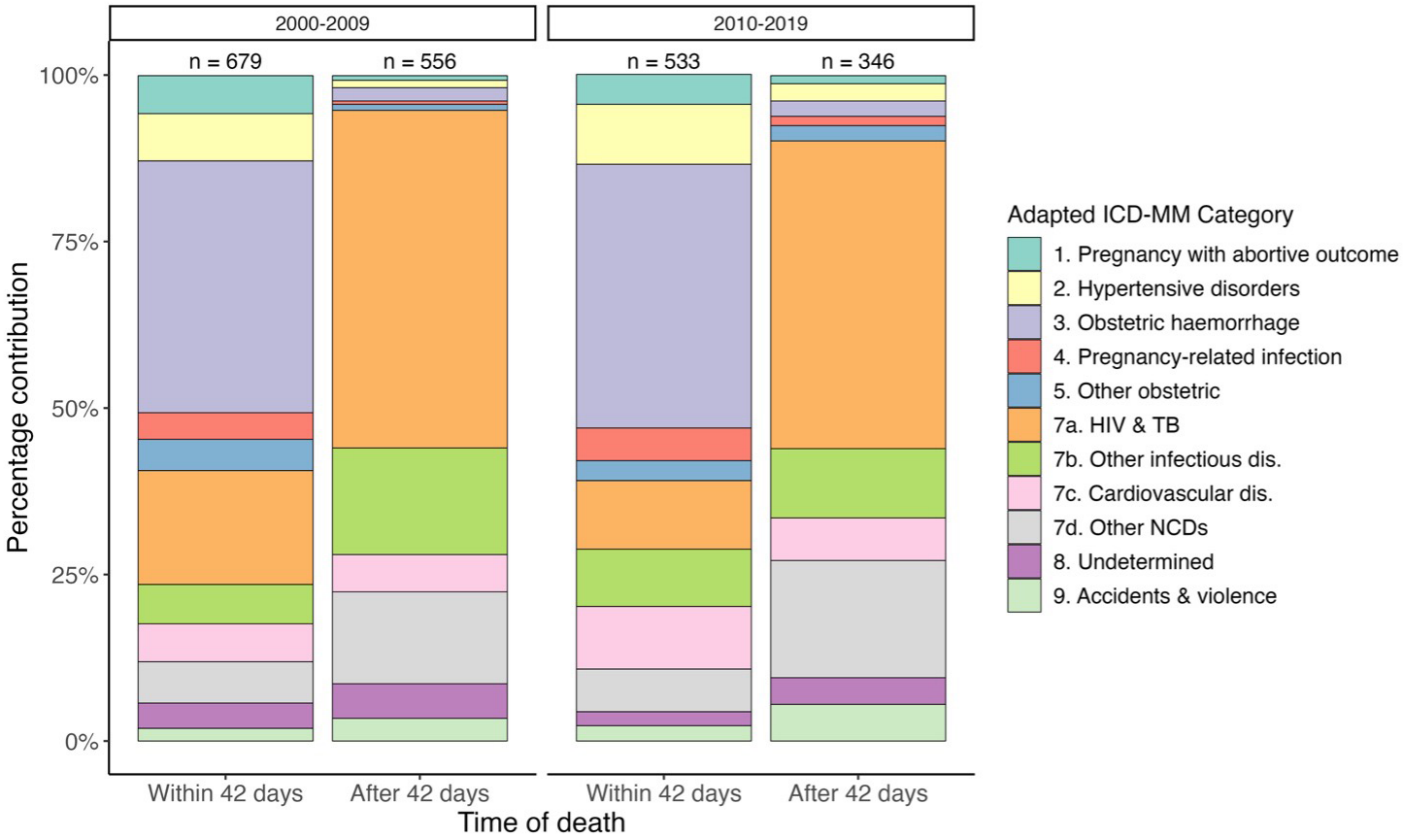
Cause of pregnancy‐related deaths up to 1 year postpartum from 2000–2009 and 2010–2019, InterVA5. *Note*: There were no deaths attributed to ICD‐MM category 6. There were unanticipated complications of management, so this category is not shown.

Figure 2 shows the causes of pregnancy‐related deaths from 2000–2009 and 2010–2019 for InterVA5. The CSMFs are similar across both time periods; however, univariable analyses suggest a slight decrease in HIV and TB, and a marginal increase in hypertensive and cardiovascular causes of deaths within 42 days. For deaths from 43 days to 1 year postpartum, univariable analyses indicate a slight decrease in other infectious diseases and increase in other NCDs. However, after accounting for HDSS heterogeneity, no changes in the predicted proportions were significant at 95%. Multinomial results for the differences by time period (2000–2009 and 2010–2019) are available in Tables S3 and S4, and Figures S5 and S6.

### 3.2 Causes of pregnancy‐related deaths by HDSS

**FIGURE 3:**
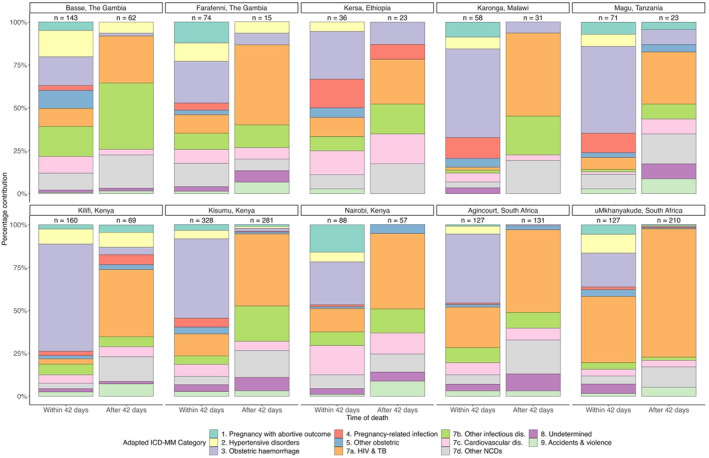
Cause of pregnancy‐related deaths up to 1 year postpartum by timing and HDSS, InterVA5. *Note*: There were no deaths attributed to ICD‐MM category 6. There were unanticipated complications of management, so this category is not shown.

Figure 3 shows substantial heterogeneity in the causes of pregnancy‐related deaths between HDSS for InterVA5. Within 42 days postpartum, obstetric haemorrhage was the leading cause of death for all sites except Basse, The Gambia and uMkhanyakude, South Africa, where other infectious diseases and HIV and TB were dominant, respectively. For deaths beyond 42 days postpartum, HIV and TB were the leading causes of death in all HDSS except Basse, The Gambia, though the contribution was greatest in Southern Africa. After adjustment for time period, multinomial predicted proportions indicate significant (non‐overlapping CIs) in the CSFM between HDSS for all causes of pregnancy‐related death. Full multinomial results for HDSS are available in Table S5 and Figure S7.

### 3.3 Circumstances of Mortality categories (COMCATs)

**FIGURE 4:**
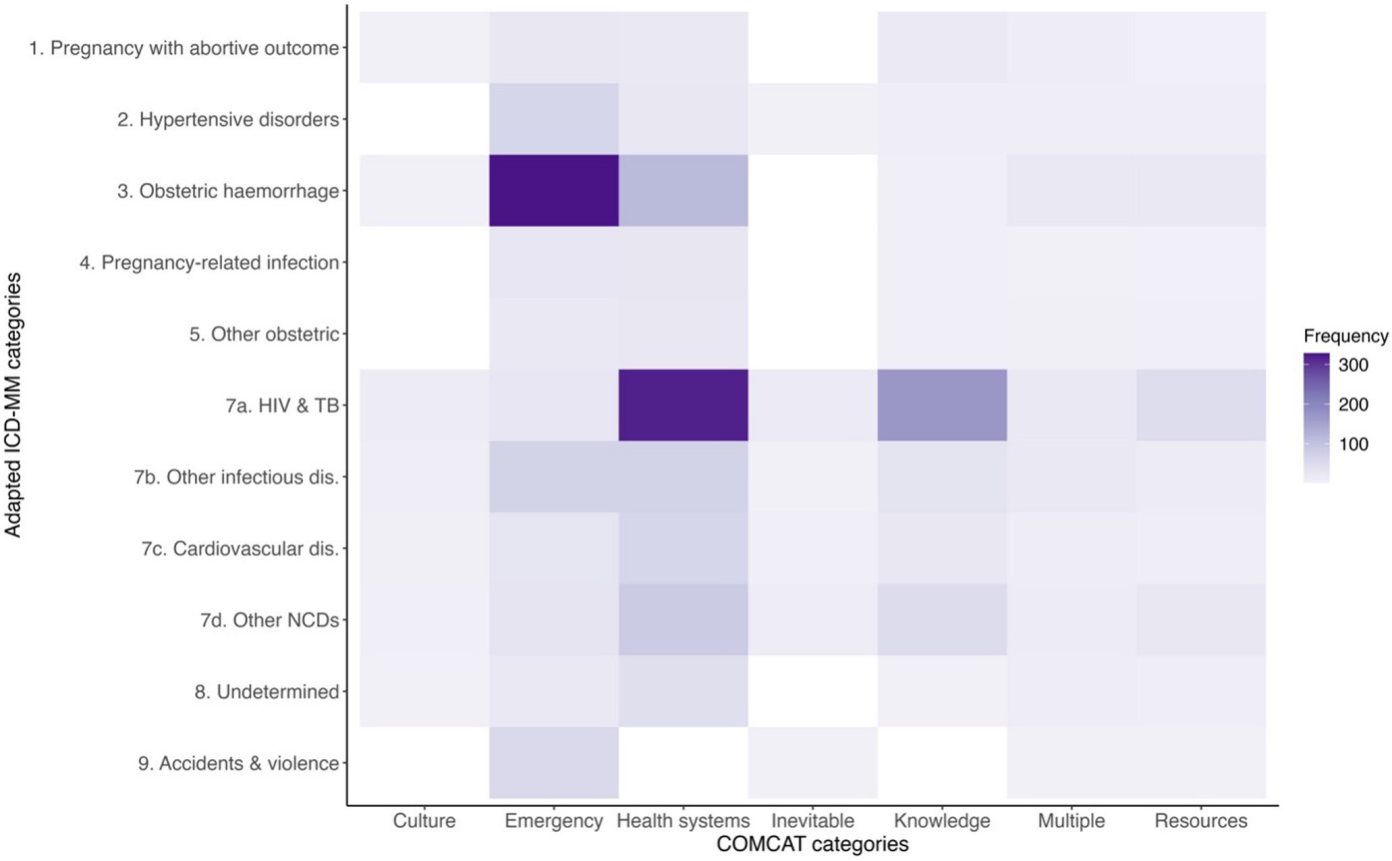
Circumstances of Mortality (COMCATs) by adapted ICD‐MM category, InterVA5. *Note*: There were no deaths attributed to ICD‐MM category 6. There were unanticipated complications of management, so this category is not shown.

Figure 4 shows that hypertensive and haemorrhagic disorders, as well as other infectious diseases, were most frequently emergencies. Deaths from HIV and TB were most frequently related to health system failures (difficulty in receiving care and adhering to treatment) and knowledge factors (lack of recognition of the severity or seriousness of disease). Cardiovascular and other NCDs were mostly related to health systems. As a result, more deaths within 42 days were emergencies, and more deaths from 43 days to 1 year postpartum were related to health system failures and knowledge (Figure S8).

## 4 DISCUSSION

### 4.1 Main findings

To our knowledge, this is the first study to compare CODs for women who died during pregnancy and within 42 days postpartum with those who died between 43 and 365 days postpartum for multiple countries in sub‐Saharan Africa.

Our results indicate the important role of infectious diseases in pregnancy‐related mortality up to 1 year postpartum. For all 10 HDSS pooled together, HIV and TB were the second largest COD occurring within 42 days and were the dominant cause for deaths occurring from 43 days to 1 year postpartum. Though also important causes of pregnancy‐related mortality within 42 days postpartum, deaths from HIV and TB, other infectious diseases and other NCDs constituted a significantly higher proportion of late pregnancy‐related deaths beyond 42 days (versus within 42 days), and health system failures were important in the circumstances of those deaths. These results corroborate the limited existing research on the causes of pregnancy‐related deaths within 42 days postpartum^17^, ^18^, ^19^ and late pregnancy‐related deaths beyond 42 days in sub‐Saharan Africa.^2^, ^3^, ^4^, ^5^ Women's repeated contacts with the health system throughout pregnancy and postpartum provides multiple opportunities to optimise management of infectious and non‐communicable diseases. New strategies may require an improved health system approach and include better training of midwives and obstetricians to identify and treat non‐obstetric conditions, better integration of non‐obstetric care within maternity, postpartum and extended postpartum care^18^, ^20^ and improved referral pathways between obstetric and non‐obstetric care‐providers. While obstetric and non‐obstetric causes of pregnancy‐related mortality remain high, African health systems require the capacity and preparedness to respond to both types of maternal health challenges simultaneously.

Our results highlight significant inter‐ and intra‐country heterogeneity between the 10 HDSS, across six countries with different underlying epidemiology and health systems. For deaths within 42 days, obstetric haemorrhage was the cause of over half of all deaths in Karonga (Malawi), Kisesa (Tanzania) and Kisumu (Kenya), compared with less than a quarter of deaths in uMkhanyakude (South Africa). HIV and tuberculosis were the leading causes only in uMkhanyakude HDSS. For deaths beyond 42 days, HIV and tuberculosis were the leading causes in all HDSS except for Basse (The Gambia) and accounted for over three‐quarters of deaths in uMkhanyakude (South Africa). In Basse, other infectious diseases were the leading cause of deaths from 43 days to 1 year postpartum. This heterogeneity highlights an urgent need for more data on causes of pregnancy‐related deaths up to 1 year postpartum across more countries in sub‐Saharan Africa to inform preventative strategies and policy.

For all 10 HDSS pooled, there were no significant differences in the causes of pregnancy‐related mortality between 2000–2009 and 2010–2019. This finding corroborates existing evidence that the causes of maternal mortality are slow to change over time,^23^ and the obstetric transition theory which hypothesises that causes are similar until the MMR falls below 50 per 100 000 live births.^21^ For pregnancy‐related deaths within 42 days and from 43 days to 1 year postpartum, there were no significant declines in the contribution of HIV and TB, despite expansions in access to ART and reductions in mother‐to‐child transmission in sub‐Saharan Africa.^22^ This may be due to one or more of the following reasons: (i) true persistence of HIV and TB relative to other causes: the proportion of deaths from HIV and TB will not decline if other causes fall more quickly. The high contribution of HIV and TB to pregnancy‐related mortality up to 1 year postpartum also corroborates existing VA evidence on deaths within six months postpartum in sub‐Saharan Africa in the post ART‐era^17^; (ii) overestimation of HIV and TB in 2010–2019: unlikely, as other evidence suggests VA algorithms may even underestimate HIV and TB‐related mortality^24^, ^25^ and misclassification would affect both 2000–2009 and 2010–2019 periods; (iii) a lack of power to detect a true change over time: power was limited by the imbalance of the sample biased towards the 2000–2009 period, especially for deaths occurring beyond 42 days postpartum. Potential explanations for this imbalance include: the time period of data contributed by the larger HDSS (e.g. Kisumu up to 2013 only); declines in VA coverage over time in some HDSS (Figure S9) and/or changes in the selection of deaths investigated with a verbal autopsy interview that prioritised deaths to recently pregnant women; or declines in mortality levels for late pregnancy‐related deaths.

While recognising these competing explanations for the persistence of HIV and TB, COMCATs emphasise the contribution of health system factors (difficulty in receiving care and adhering to treatment) and knowledge factors (lack of recognition of the severity or seriousness of disease). These findings suggest ANC and PNC programmes in high‐prevalence contexts require more emphasis on facilitating treatment adherence during pregnancy and postpartum, and also echo earlier calls for complication readiness programmes to include postpartum monitoring of HIV‐positive women,^26^ which – for some high‐risk women – should extend beyond 42 days.^8^

Consistent with other research^16^, ^27^ our analysis reveals low concordance between physician‐assigned and algorithm‐determined underlying cause of death and adapted ICD‐MM category, though concordance is high for the type of death (obstetric or non‐obstetric). Unlike the algorithms, physicians can use the VA narrative report that describes the sequencing of events that led to death to help attribute an underlying cause, but the quality of training affects the accuracy of physician‐coded VA. Previous research has demonstrated 50% or lower concordance of physician‐coded VA with hospital‐based autopsy.^28^

Finally, from VA data alone, it is not possible to identify which non‐obstetric deaths were maternal (i.e. were aggravated by pregnancy). Our results emphasise the urgent need for better linkage and triangulation of data sources so that maternal deaths can be accurately identified and classified in sub‐Saharan Africa.^10^

### 4.2 Strengths and limitations

A strength of this study is the large sample size of deaths during pregnancy and up to 1 year postpartum across 10 HDSS in six different African countries. The use of VA data for deaths that occur in the community, moreover, means our findings on the COD distribution during pregnancy and the extended postpartum period are more generalisable to the communities where COD attribution is most urgently needed. We also evaluated COD by broad type and adapted ICD‐MM categories, and by InterVA5, InSilicoVA and physician review where available.

The limitations of this study are threefold. First, the time period covered is broad, and substantial changes in the burden of disease and health system responses occurred throughout this period that were beyond the scope of this study to explore in detail. Secondly, COD may be misclassified. Both InterVA5 and InSilicoVA algorithms have a weighting factor that make attribution of an obstetric cause more likely if the death occurred within 42 days. Our results may therefore overestimate obstetric causes within 42 days and underestimate obstetric causes after 42 days postpartum. Thirdly, HDSS data are often incomplete; VA interviews were not completed for all reproductive‐age deaths in every HDSS (Table S6), and not all pregnancies and births were recorded in the delivery file – e.g. early pregnancy outcomes such as ectopic pregnancy, miscarriage or abortion were only recorded in the delivery file in four HDSS. Across all sites, recording of stillbirths may be incomplete if stigma or cultural taboos precluded disclosure of the birth to enumerators. Some pregnancy‐related deaths may also have been missed if respondents did not report that the deceased was recently pregnant during the VA interview and if the algorithm did not identify signs or symptoms of recent pregnancy. These problems of misclassification and incompleteness may bias our results.

### 4.3 Interpretation

Despite the difficulty in attribution, available evidence indicates the potential plausibility of women's heightened vulnerability to infectious disease beyond 42 days postpartum. Postpartum immune recovery and its effect on susceptibility to infectious disease is poorly understood, but normal cellular function may take 3–4 months to recover.^29^ Sleep deprivation, lactation and recovery from labour and delivery drive a pro‐inflammatory state^30^ or ‘immune reconstitution’, which might contribute to an exacerbation of latent infection in the postpartum period. In sub‐Saharan Africa in particular, the high prevalence of anaemia among women of reproductive age^31^ might further increase susceptibility to infectious disease.

There is a need for further research into women's vulnerability to infectious disease (and co‐infection) during the extended postpartum period.^18^ Though evidence is scarce, coincidental deaths from HIV during the postpartum period might be less likely because very sick women with advanced HIV infection are unlikely to get pregnant.^32^ Rather, pregnancy might accelerate HIV progression, or HIV might cause heightened vulnerability to other postpartum complications. In the WHO maternal mortality estimation models, the fraction of pregnancy‐related HIV deaths assumed to be HIV‐related indirect maternal deaths (i.e. not coincidental) is 0.3.^7^ There is an urgent need to update the evidence for this assumption and, in particular, its applicability to pregnancy‐related deaths beyond 42 days postpartum. Pregnancy and postpartum recovery may also aggravate tuberculosis (co‐)infection, through an acute worsening of active TB or an exacerbation of latent infection.^19^, ^33^ An increased risk of active TB may continue up to 6 months postpartum. Finally, available evidence on the women's susceptibility to malaria infection postpartum is contradictory^34^, ^35^ and even less is known about susceptibility to infection or its severity in the extended postpartum period.

## 5 CONCLUSION

Infectious diseases are the predominant cause of late pregnancy‐related deaths from 43 to 365 days postpartum, followed by NCDs, in Kenya, The Gambia, Malawi, Tanzania, Ethiopia and South Africa, and this was the case in both time periods (2000–2009 and 2010–2019). Further research is urgently required to understand women's potentially heightened vulnerability to infectious and non‐communicable diseases in the extended postpartum period.

## AUTHOR CONTRIBUTIONS

UG (guarantor): Conceptualisation, methodology, formal analysis, visualisation, writing – original draft. GR: Conceptualisation, methodology, writing – review and editing. JERP: Formal analysis, methodology, writing – review and editing. CC: Methodology, writing – review and editing. MJ, KH, SK, DO, DK, AD, MD, CWK, KK, MU, AN: Data acquisition & preparation, writing – review and editing. MT: Clinical interpretation, writing – review and editing. LAM: Clinical interpretation, methodology, writing – review and editing; PvD: Clinical interpretation, writing – review and editing. VF: Conceptualisation, methodology, interpretation, writing – review and editing.

## FUNDING INFORMATION

This study was supported by the UKRI Economic and Social Research Council as the funders of UG PhD studentship (grant reference ES/P000592/1). Each HDSS is individually funded and donor agencies vary. The Basse and Farafenni HDSS are supported by UKRI‐MRC. uMkhanyakude (AHRI) HDSS is supported by the DSI‐MRC South African Population Research Infrastructure Network (SAPRIN) and the Wellcome Trust. The Karonga HDSS (MEIRU) is funded by the Wellcome Trust. The Nairobi HDSS is funded by the Rockefeller Foundation and the Wellcome Trust. The Kisumu HDSS (KEMRI/CDC) is funded by PEPFAR and the President's Malaria Initiative (PMI) from the U.S. Centres of Disease Control and Prevention (CDC). Kilifi HDSS (KEMRI‐Wellcome) is funded by the Wellcome trust and receives project‐specific funding from MEASURE and the Gavi Alliance. The MRC/Wits Agincourt HDSS receives core funding from the South African Research Council and the Department of Science and Innovation, SA, as well as international funding from the Wellcome Trust. The Kersa HDSS is funded by the CDC and the Ethiopian Public Health Authority. The Magu HDSS has most recently been funded by the Wellcome Trust.

## CONFLICT OF INTEREST STATEMENT

None declared.

## ETHICS APPROVAL

This study was approved by the London School of Hygiene and Tropical Medicine ethical review committee (reference 26603, original approval date 13 December 2021; amendment approval date 5 June 23).

## Supporting information


Appendix S1.

## Data Availability

Each HDSS manages access to their microdata. Prospective users can apply for access to data for Karonga, Kilifi, Nairobi and uMkhanyakude from the HDSS online repositories and contact site managers at the remaining HDSS to request access. The data are not publicly available due to privacy or ethical restrictions.
