# Supplementary material for: Pregnancy‐related mortality up to 1 year postpartum in sub‐Saharan Africa: an analysis of verbal autopsy data from six countries
Source: BJOG. 2023 Jul 19;131(2):163–74. doi: 10.1111/1471-0528.17606 (PMC10952650; doi:10.1111/1471-0528.17606)

**Supplementary Material 1**

**Pregnancy-related mortality up to one year postpartum in sub-Saharan Africa: an analysis of verbal autopsy data from six countries**

Ursula Gazeley,^1,2^ Georges Reniers,^2^ Julio Romero Prieto,^2^ Clara Calvert,^1,3^ Momodou Jasseh,^4^ Kobus Herbst,^5,6^ Sammy Khagayi,^7^ David Obor,^7^ Daniel Kwaro, ^7^ Albert Dube,^8^ Merga Dheresa,^9^ Chodziwadziwa W. Kabudula,^10^ Kathleen Kahn,^10,11^ Mark Urassa,^12^ Amek Nyaguara,^13^ Marleen Temmerman,^14^ Laura A. Magee,^15,16^ Peter von Dadelszen,^15,16^ and Veronique Filippi^1^

**Affiliations**

^1^ Department of Infectious Disease Epidemiology, London School of Hygiene and Tropical Medicine, UK

^2^ Population Studies Group, London School of Hygiene and Tropical Medicine, UK

^3^ Usher Institute, University of Edinburgh, Edinburgh, UK

^4^ Medical Research Council Unit The Gambia at LSHTM, The Gambia

^5^ Africa Health Research Institute, Durban, South Africa

^6^ DSI-MRC South African Population Research Infrastructure Network (SAPRIN), Durban, South Africa.

^7^ Kenya Medical Research Institute – Centre for Global Health Research, Kisumu, Kenya

^8^ Malawi Epidemiology and Intervention Research Institute, Karonga, Malawi

^9^  School of Nursing and Midwifery, College of Health and Medical Sciences, Haramaya University, Harar, Ethiopia

^10^ MRC/Wits Rural Public Health and Health Transitions Research Unit (Agincourt), School of Public Health, Faculty of Health Sciences, University of the Witwatersrand, Johannesburg, South Africa

^11^Department of Epidemiology and Global Health, Umeå University, Umeå, Sweden

^12^ The Tazama Project, National Institute for Medical Research, Mwanza, Tanzania

^13^ KEMRI-Wellcome Trust, Kilifi, Kenya

^14^ Centre of Excellence in Women and Child Health, Aga Khan University. Nairobi, Kenya

^15^ Department of Women and Children's Health, School of Life Course and Population Sciences, Faculty of Life Science and Medicine, Kings College London, London, UK

^16^ Institute of Women and Children’s Health, King’s College London, UK.

# Abstract

The main paper classifies causes of death according to an adapted version of the ICD-MM categories, such that they could be applied to the analysis of pregnancy-related mortality. In Section A, we justify this adaptation and explain the mapping from the ICD-MM to our adapted categories.

In Section B, we display the timing of 63 deaths that InterVA5 attributed to obstetric causes which occurred beyond 42 days postpartum. For obstetric haemorrhage in particular, analysis of the postpartum timing of death suggests women may die of delayed or ongoing consequences up to 100 days postpartum.

In Section C, we present the multinomial results for the main InterVA5 results, adjusting for HDSS and decade (2000-2009 and 2010-2019).

In Sections D & E we present two sensitivity analyses. First, we present the main results using an alternative algorithm, InSilicoVA. We show that the main results are not sensitive to the choice of the algorithm, and the main findings are consistent between algorithms. We also present concordance in the cause of death for InterVA5, InSilicoVA and physician-assigned cause of death. We show that concordance of both algorithms with physician-coded VA is high for the type of death (obstetric, non-obstetric, undetermined, external), but lower for ICD-MM category, and lower still for underlying cause of death. InterVA slightly outperforms InSilicoVA. Concordance between the two algorithms is high.

In Section F we present the results using InterVA5 for the two additional predictors – the decade the death occurred in (2000-2009 and 2010-2019) and the HDSS. We show that there have been no significant changes in the predicted proportions of 11-cause categories by decade when stratified by the time of death. We also show that there is significant heterogeneity by HDSS, with the cause-specific mortality fractions for the 11-cause categories varying between HDSS.

In Section G we present the breakdown of pregnancy-related deaths by COMCAT categories. For deaths occurring after (vs. within) 42 days postpartum, fewer were emergencies and more related to either problems receiving care in health systems, or to knowledge, recognition, or awareness of serious disease.

Finally, in Section H we present the coverage of VA data for all deaths in eight of the ten HDSS with available data. While coverage is complete in three HDSS (Nairobi, Karonga, and uMkhanyakude), it is incomplete in the five HDSS (Agincourt, Basse, Farafenni, Kisumu and Magu). Coverage in these HDSS varies by year.

Table of Contents

[Abstract 1](#_Toc136620446)

[A. Adapted ICD-MM categories applied to pregnancy-related mortality 4](#_Toc136620447)

[B. Obstetric deaths beyond 42 days postpartum 5](#_Toc136620448)

[C. Multinomial predicted proportions for the main results, InterVA5 6](#_Toc136620449)

[D. Replication of the main results with InSilicoVA 7](#_Toc136620450)

[E. Concordance between algorithm- and physician-assigned cause of death 9](#_Toc136620451)

[F. Multinomial predicted proportions for the additional predictors, InterVA5 10](#_Toc136620452)

[Decade 10](#_Toc136620453)

[Health and Demographic Surveillance Systems 12](#_Toc136620454)

[G. Circumstances of Mortality Categories (COMCATs) 16](#_Toc136620455)

[F . Verbal autopsy coverage by HDSS 17](#_Toc136620456)

Table of Figures

[**Figure S1 Mapping of adapted ICD-MM categories** 4](#_Toc136620462)

[**Figure S2 Late obstetric deaths by postpartum day of death** 5](#_Toc136620463)

[**Figure S3 Predicted proportion results by ICD-MM category and timing, InterVA5** 6](file:////Users/ursulagazeley/Library/CloudStorage/GoogleDrive-ursulagazeley@gmail.com/My%20Drive/PhD/1.%20HDSS%20/3.%20Verbal%20autopsy%20/Manuscript/BJOG/Revised/Gazeley_Appendix_BJOG_REVISED_020623.docx#_Toc136620464)

[**Figure S4 Cause of pregnancy-related deaths up to one year postpartum by timing, age, and type of cause, InSilicoVA** 8](#_Toc136620465)

[**Figure S5 Multinomial regression predicted proportions for deaths within 42 days postpartum by decade, InterVA5** 11](#_Toc136620466)

[**Figure S6 Multinomial regression predicted proportions for deaths after 42 days postpartum by decade, InterVA5** 12](#_Toc136620467)

[**Figure S7 Multinomial regression predicted proportions for deaths after 42 days postpartum by HDSS, InterVA5** 15](#_Toc136620468)

[**Figure S8 COMCATs by time of death, InterVA5** 16](#_Toc136620469)

[**Figure S9 Verbal autopsy coverage for deaths by year** 18](file:////Users/ursulagazeley/Library/CloudStorage/GoogleDrive-ursulagazeley@gmail.com/My%20Drive/PhD/1.%20HDSS%20/3.%20Verbal%20autopsy%20/Manuscript/BJOG/Revised/Gazeley_Appendix_BJOG_REVISED_020623.docx#_Toc136620470)

Table of Tables

[**Table S1 Predicted proportion results by ICD-MM category and timing, InterVA5** 6](#_Toc136620471)

[**Table S2 Agreement between physician-assigned and algorithm-assigned cause of death** 9](#_Toc136620472)

[**Table S3 Predicted margins results by ICD-MM category and decade, InterVA5, deaths within 42 days** 10](#_Toc136620473)

[**Table S4 Predicted margins results by ICD-MM category and decade, InterVA5, deaths after 42 days** 11](#_Toc136620474)

[**Table S5 Predicted margins results by ICD-MM category and HDSS, InterVA5** 13](#_Toc136620475)

[**Table S6 Coverage of VA data by HDSS** 17](#_Toc136620476)

# Adapted ICD-MM categories applied to pregnancy-related mortality

We grouped deaths according to the four types and nine adapted International Classification of Diseases-Maternal Mortality (ICD-MM) categories as follows: *Obstetric* (1. Pregnancy with abortive outcome, 2. Hypertensive disorders, 3. Obstetric haemorrhage, 4. Pregnancy-related infection, 5. Other obstetric complications, 6. Unanticipated complications of management); *Non-obstetric* (7a. HIV and tuberculosis, 7b. Other infectious diseases, 7c. Cardiovascular diseases, 7d. Other NCDs); *Unspecified* (8. Undetermined); and *External* (9. Accidental). Exact replication of the ICD-MM categories was not possible because we analysed pregnancy-related deaths (i.e. defined only by time of death), and not maternal mortality. From VA data alone, it is not possible to differentiate which non-obstetric pregnancy-related deaths were indirect maternal and which were coincidental; this would require a clinical COD expert reviewing a patients’ medical records to ascertain whether the underlying condition (e.g., HIV, carcinoma, or cardiovascular disease) was “aggravated by pregnancy” – as is required for the death to be considered maternal. Without further record linkage and data source triangulation this was not possible, and hence we modified the ICD-MM categories to apply to pregnancy-related mortality (see Figure S1 below).

*Obstetric* and *Unspecified* groups replicate the ICD-MM categories; *Non-obstetric* includes all non-obstetric causes without an attribution whether the death was indirect maternal or coincidental to the pregnancy; *External* includes deaths from accidents and violent injuries only.

**Figure S1 Mapping of adapted ICD-MM categories**


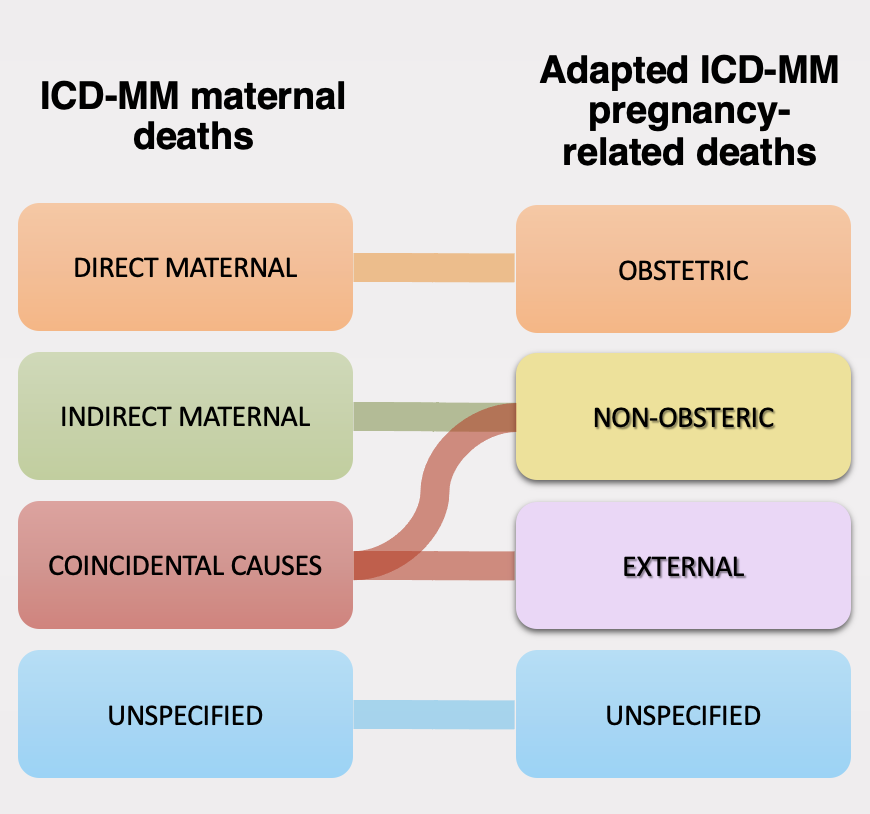


# Obstetric deaths beyond 42 days postpartum

Most direct obstetric deaths occur very shortly after delivery or pregnancy termination. In total, we identified 63 deaths that HDSS and verbal autopsy data suggest occurred beyond 42 days postpartum and from a direct obstetric cause. Figure S2 shows the timing of these deaths by days postpartum.

There are several potential explanations for these causes occurring so late postpartum:

1. Delayed effects of the obstetric complication. A woman’s death may have been prevented for the standard postpartum period but she may die later on after prolonged illness.
2. The obstetric death may relate to a repeat pregnancy, not the index pregnancy recorded in the HDSS data or the verbal autopsy data. If this is the case, these are deaths during pregnancy and within 42 days, and not late pregnancy-related deaths.
3. Incorrect date of death, date of delivery, or cause of death.

Though only speculative, the concentration of deaths shortly after 42 days from obstetric haemorrhage, hypertensive disorders, and pregnancy-related infection, may suggest explanation 1, with some women may die from prolonged illness.

For all obstetric causes, deaths occurring very late on may suggest explanation 2, with enough time elapsed for a woman to have become pregnant again, e.g. pregnancy with abortive outcome at day 300 postpartum may relate to pregnancy n+1.

**Figure S2 Late obstetric deaths by postpartum day of death**


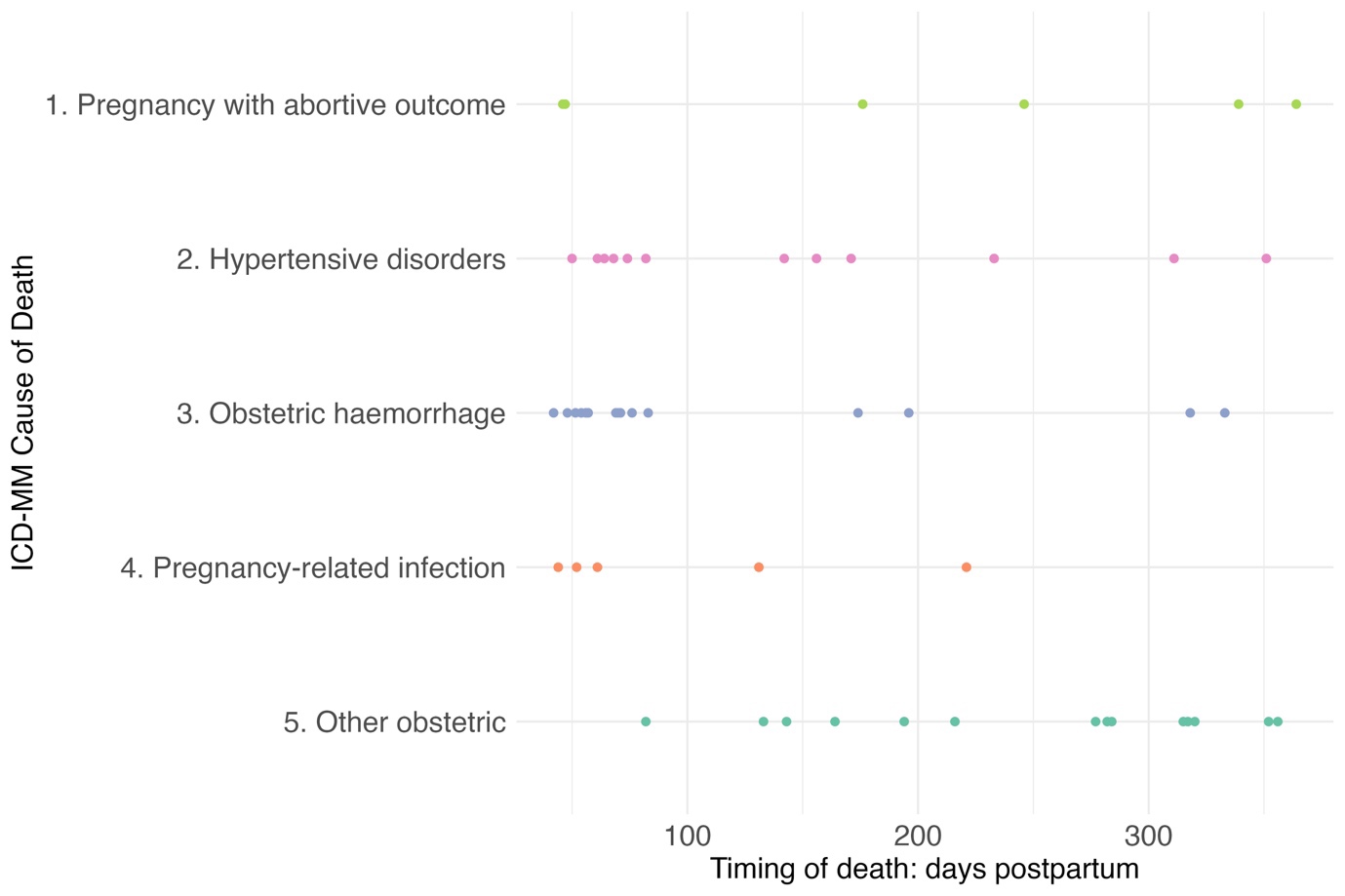


# Multinomial predicted proportions for the main results, InterVA5

Table S1 presents the full predicted margins results for the multinomial regression for the proportion of deaths in each ICD-MM category by timing for InterVA5. These results are shown graphically in Figure S3 below.

**Table S1 Predicted proportion results by ICD-MM category and timing, InterVA5**

| **Adapted ICD-MM Category^a^** | **Timing** | **Margin** | **SE^b^** | **p-value** | **Lower CI** | **Upper CI** |
| --- | --- | --- | --- | --- | --- | --- |
| 1. **Pregnancy with abortive outcome** | After 42 days | 0.010 | 0.004 | 0.005 | 0.003 | 0.017 |
|  | Within 42 days | 0.050 | 0.006 | 0.000 | 0.038 | 0.062 |
| 1. **Hypertensive disorders** | After 42 days | 0.019 | 0.005 | 0.000 | 0.010 | 0.029 |
|  | Within 42 days | 0.076 | 0.008 | 0.000 | 0.061 | 0.091 |
| 1. **Obstetric haemorrhage** | After 42 days | 0.024 | 0.005 | 0.000 | 0.013 | 0.034 |
|  | Within 42 days | 0.377 | 0.014 | 0.000 | 0.350 | 0.403 |
| 1. **Pregnancy-related infection** | After 42 days | 0.010 | 0.004 | 0.004 | 0.003 | 0.018 |
|  | Within 42 days | 0.041 | 0.006 | 0.000 | 0.030 | 0.052 |
| 1. **Other direct obstetric** | After 42 days | 0.016 | 0.004 | 0.000 | 0.007 | 0.024 |
|  | Within 42 days | 0.039 | 0.006 | 0.000 | 0.028 | 0.050 |
| **7a. HIV & TB** | After 42 days | 0.454 | 0.016 | 0.000 | 0.422 | 0.486 |
|  | Within 42 days | 0.160 | 0.011 | 0.000 | 0.140 | 0.181 |
| **7b. Other infectious diseases** | After 42 days | 0.152 | 0.012 | 0.000 | 0.128 | 0.176 |
|  | Within 42 days | 0.067 | 0.007 | 0.000 | 0.054 | 0.081 |
| **7c. Cardiovascular diseases.** | After 42 days | 0.062 | 0.008 | 0.000 | 0.046 | 0.078 |
|  | Within 42 days | 0.073 | 0.008 | 0.000 | 0.059 | 0.088 |
| **7d. Other NCDs** | After 42 days | 0.162 | 0.013 | 0.000 | 0.137 | 0.187 |
|  | Within 42 days | 0.063 | 0.007 | 0.000 | 0.049 | 0.077 |
| **8. Undetermined** | After 42 days | 0.047 | 0.007 | 0.000 | 0.033 | 0.061 |
|  | Within 42 days | 0.032 | 0.005 | 0.000 | 0.022 | 0.042 |
| **9. Accidents & violence** | After 42 days | 0.043 | 0.007 | 0.000 | 0.030 | 0.057 |
|  | Within 42 days | 0.022 | 0.004 | 0.000 | 0.013 | 0.030 |
| **^a^** There were no deaths in the data attributed to category 6. Unanticipated complications of management, so not shown.  **^b^**  Standard error | | | | | | |

**Figure S3 Predicted proportion results by ICD-MM category and timing, InterVA5**


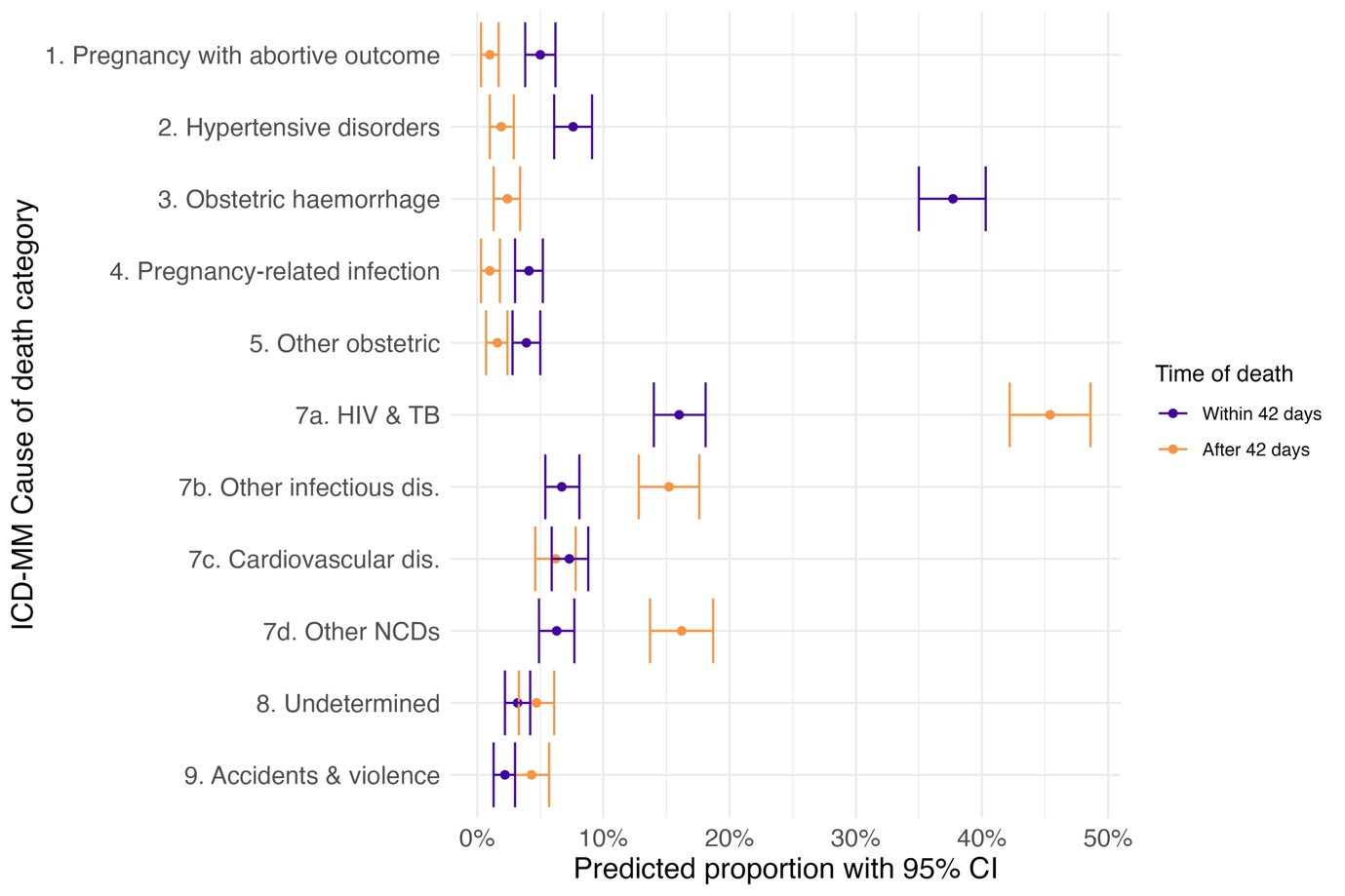


# Replication of the main results with InSilicoVA

Figure S4 panel A shows the proportion of deaths for three categories of pregnancy-related death: obstetric, non-obstetric, and external. Unlike InterVA5, InSilicoVA does not assign “undetermined” cause, and hence the fourth category is not represented in Figure S2. However, consistent with the InterVA5 results, across all age groups, obstetric deaths are dominant for deaths occurring within 42 days, while non-obstetric deaths are dominant for deaths occurring beyond 42 days postpartum. External deaths from accidental causes comprise a small proportion of the deaths.

Figure S4 panel B shows the breakdown of these three cause groupings by the ICD-MM categories. Category 7 – non-obstetric deaths – are disaggregated by subgroup: a) HIV and tuberculosis, b) other infectious diseases, c) cardiovascular diseases, d) other non-communicable diseases (NCDs). Consistent with the InterVA5 results, for deaths occurring within 42 days, obstetric haemorrhage is the dominant cause of obstetric deaths, and HIV and tuberculosis are the dominant causes of non-obstetric deaths. For late pregnancy-related deaths occurring beyond 42 days but within one year, HIV and TB are the leading causes, followed by other infectious diseases, and other NCDs.

ICD-MM category 6 for unanticipated complications of management is missing because there were no deaths in this category in this pooled sample.

**Figure S4 Cause of pregnancy-related deaths up to one year postpartum by timing, age, and type of cause, InSilicoVA**


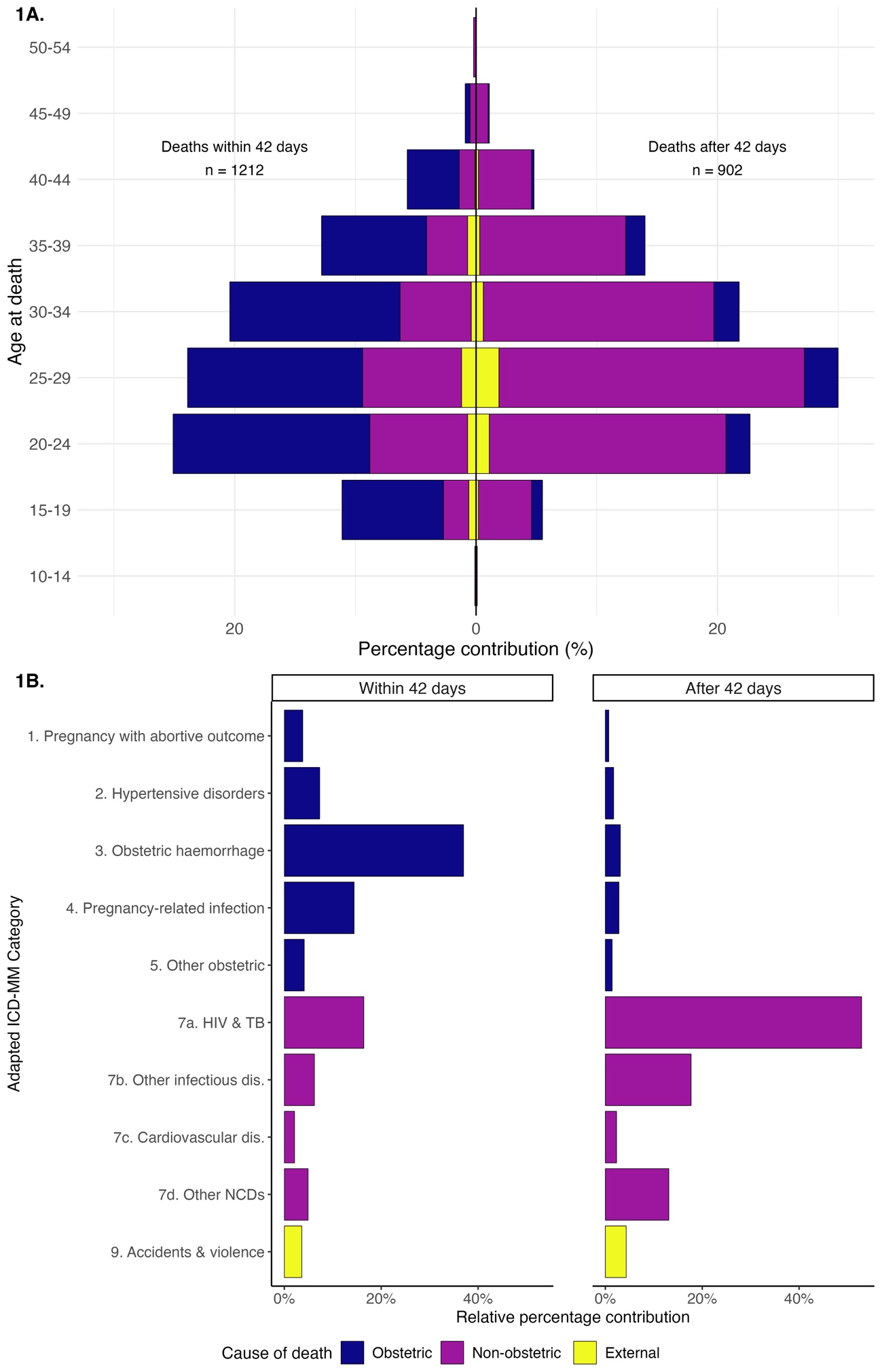


^Note: There were no deaths attributed to ICD-MM category 6. Unanticipated complications of management so this category is not shown. InSilicoVA does not have a category for 8. Unspecified, so not shown.^

# Concordance between algorithm- and physician-assigned cause of death

The percent concordance for each HDSS and algorithm is shown in Table S2. Concordance was assessed as agreement in the string (allowing for spelling differences) in the underlying cause of death, adapted ICD-MM category, and broad type. Physician-assigned cause of death data were only available for three HDSS: Kisumu and Nairobi (Kenya), and Karonga (Malawi). For all ten HDSS, we calculated the concordance between InterVA5 and InSilicoVA. Concordance of either algorithm with physician-assigned underlying cause of death was low, but slightly higher for InterVA5. Across all HDSS, concordance between the algorithms is much higher than concordance with physician-assigned causes. Concordance of InterVA5 with physician-coded VA slightly outperforms InSilicoVA. Since hospital-based deaths are the reference standard, it is not clear which method determined the true underlying cause of death.

**Table S2 Agreement between physician-assigned and algorithm-assigned cause of death**

| **HDSS** | **Category** | **InterVA5 vs. Physician review** | **InSilicoVA vs. Physician review** | **InterVA5 vs. InSilicoVA** |
| --- | --- | --- | --- | --- |
| Agincourt, South Africa | Underlying cause |  |  | 69% |
|  | Adapted ICD-MM category |  |  | 72% |
|  | Type |  |  | 87% |
| Basse, The Gambia | Underlying cause |  |  | 64% |
|  | Adapted ICD-MM category |  |  | 67% |
|  | Type |  |  | 85% |
| Farafenni, The Gambia | Underlying cause |  |  | 68% |
|  | Adapted ICD-MM category |  |  | 70% |
|  | Type |  |  | 81% |
| Karonga, Malawi | Underlying cause | 32% | - 25% | 61% |
|  | Adapted ICD-MM category | 44% | - 34% | 73% |
|  | Type | 83% | - 82% | 91% |
| Kersa, Ethiopia | Underlying cause | - |  | 73% |
|  | Adapted ICD-MM category | - |  | 76% |
|  | Type | - |  | 92% |
| Kilifi, Kenya | Underlying cause | 43% |  | 79% |
|  | Adapted ICD-MM category | 55% |  | 79% |
|  | Type | 83% |  | 91% |
| Kisumu, Kenya | Underlying cause | 43% | - | 51% |
|  | Adapted ICD-MM category | 55% | - | 58% |
|  | Type | 83% | - | 87% |
| Magu, Tanzania | Underlying cause | - | - | 47% |
|  | Adapted ICD-MM category | - | - | 64% |
|  | Type | - | - | 88% |
| Nairobi, Kenya | Underlying cause | 27% | 26% | 68% |
|  | Adapted ICD-MM category | 36% | 33% | 79% |
|  | Type | 68% | 65% | 81% |
| uMkhanyakude, South Africa | Underlying cause |  |  | 78% |
|  | Adapted ICD-MM category |  |  | 84% |
|  | Type |  |  | 92% |
| **Total** | **Underlying cause** | **34%** | **26%** | **65%** |
|  | **Adapted ICD-MM category** | **45%** | **34%** | **71%** |
|  | **Type** | **78%** | **74%** | **89%** |
| Missing values are present as not all HDSS had physician-coded VA data. For Kisumu, physician-coded COD was only available for deaths with an InterVA5 VA result. | | | | |

# Multinomial predicted proportions for the additional predictors, InterVA5

## Time period (2000-2009 and 2010-2019)

Table S3 and Figure S5 show the predicted proportions from multinomial regression for InterVA5 for deaths within 42 days postpartum; Table S4 and Figure S6 show the predicted proportions for deaths from 43 days to one year postpartum. Margins were stratified by the timing of death because of differences in the cause-specific mortality fractions by timing for each decade. Using the mean of the whole sample to analyse the margins for decade may therefore obscure changes to the causes of death depending on when the death occurs. There were no significant changes in the causes of death over this time period.

**Deaths within 42 days postpartum**

**Table S3 Predicted margins results by ICD-MM category and decade, InterVA5, deaths within 42 days**

| **ICD-MM Category^a^** | **Decade** | **Margin** | **SE^b^** | **p-value** | **Lower CI** | **Upper CI** |
| --- | --- | --- | --- | --- | --- | --- |
| 1. **Pregnancy with abortive outcome** | 2000-2009 | 0.056 | 0.009 | 0.000 | 0.039 | 0.073 |
|  | 2010-2019 | 0.047 | 0.009 | 0.000 | 0.029 | 0.065 |
| 1. **Hypertensive disorders** | 2000-2009 | 0.073 | 0.010 | 0.000 | 0.053 | 0.093 |
|  | 2010-2019 | 0.086 | 0.012 | 0.000 | 0.063 | 0.109 |
| 1. **Obstetric haemorrhage** | 2000-2009 | 0.395 | 0.018 | 0.000 | 0.360 | 0.431 |
|  | 2010-2019 | 0.376 | 0.021 | 0.000 | 0.335 | 0.416 |
| 1. **Pregnancy-related infection** | 2000-2009 | 0.042 | 0.008 | 0.000 | 0.026 | 0.057 |
|  | 2010-2019 | 0.046 | 0.009 | 0.000 | 0.029 | 0.063 |
| 1. **Other direct obstetric** | 2000-2009 | 0.043 | 0.008 | 0.000 | 0.028 | 0.059 |
|  | 2010-2019 | 0.035 | 0.008 | 0.000 | 0.020 | 0.050 |
| **7a. HIV & TB** | 2000-2009 | 0.143 | 0.011 | 0.000 | 0.121 | 0.165 |
|  | 2010-2019 | 0.138 | 0.013 | 0.000 | 0.112 | 0.163 |
| **7b. Other infectious diseases** | 2000-2009 | 0.076 | 0.009 | 0.000 | 0.059 | 0.094 |
|  | 2010-2019 | 0.064 | 0.009 | 0.000 | 0.047 | 0.082 |
| **7c. Cardiovascular diseases.** | 2000-2009 | 0.063 | 0.008 | 0.000 | 0.046 | 0.080 |
|  | 2010-2019 | 0.087 | 0.011 | 0.000 | 0.065 | 0.110 |
| **7d. Other NCDs** | 2000-2009 | 0.058 | 0.008 | 0.000 | 0.043 | 0.073 |
|  | 2010-2019 | 0.069 | 0.010 | 0.000 | 0.051 | 0.088 |
| **8. Undetermined** | 2000-2009 | 0.033 | 0.006 | 0.000 | 0.021 | 0.044 |
|  | 2010-2019 | 0.026 | 0.006 | 0.000 | 0.014 | 0.038 |
| **9. Accidents & violence** | 2000-2009 | 0.017 | 0.004 | 0.000 | 0.009 | 0.025 |
|  | 2010-2019 | 0.025 | 0.006 | 0.000 | 0.013 | 0.038 |
| **^a^** There were no deaths in the data attributed to category 6. Unanticipated complications of management, so not shown.  **^b^**  Standard error | | | | | | |

**Figure S5 Multinomial regression predicted proportions for deaths within 42 days postpartum by decade, InterVA5**

***
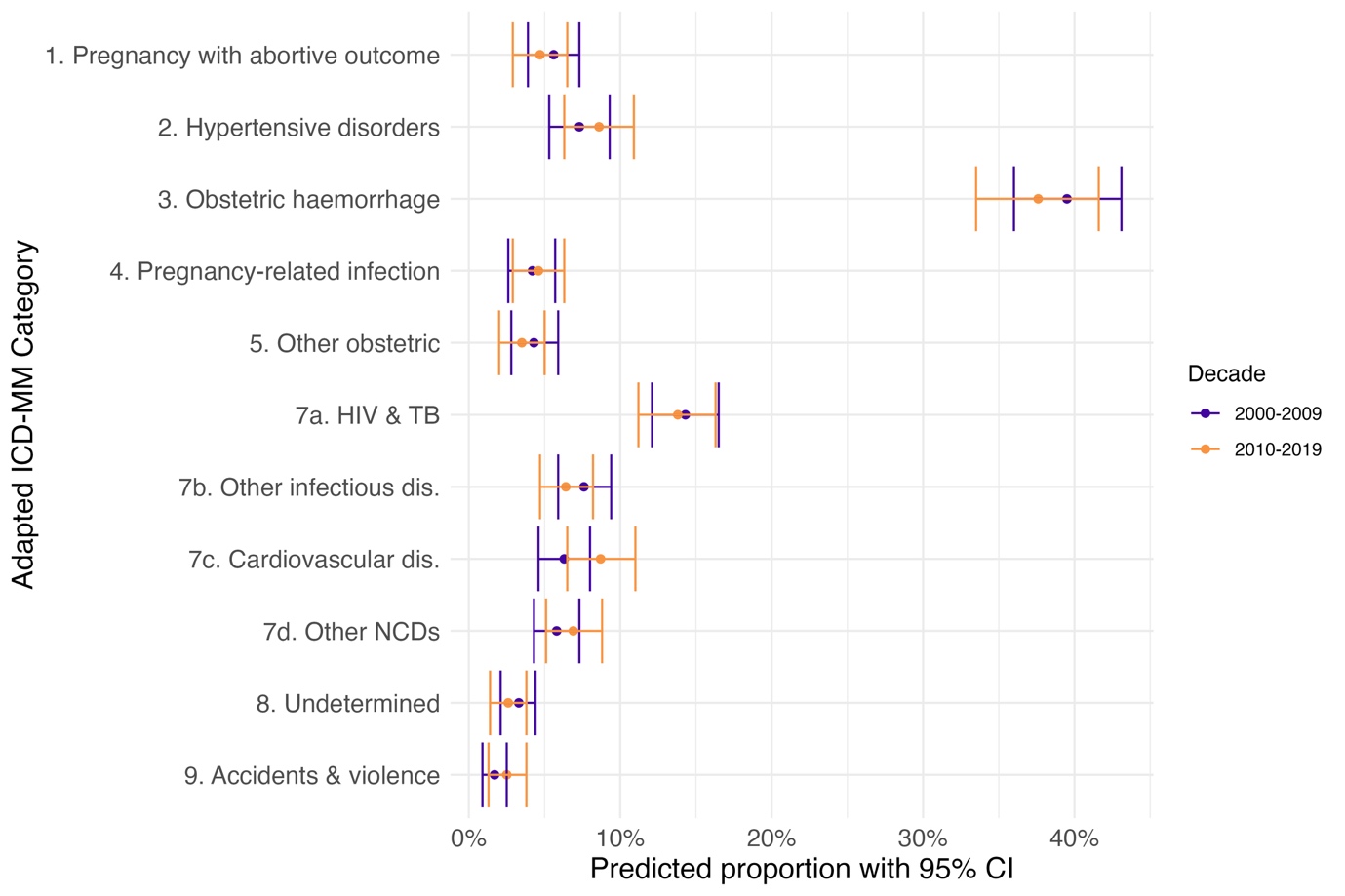
***

**Note:** InterVA attributed no deaths to category 6. Unanticipated complications of management, so not shown.

**Deaths after 42 days postpartum (43-365 days)**

**Table S4 Predicted margins results by ICD-MM category and decade, InterVA5, deaths after 42 days**

| **ICD-MM Category^a^** | **Decade** | **Margin** | **SE^b^** | **p-value** | **Lower CI** | **Upper CI** |
| --- | --- | --- | --- | --- | --- | --- |
| 1. **Pregnancy with abortive outcome** | 1998-2008 | 0.010 | 0.003 | 0.006 | 0.003 | 0.016 |
|  | 2009-2019 | 0.008 | 0.003 | 0.011 | 0.002 | 0.014 |
| 1. **Hypertensive disorders** | 1998-2008 | 0.016 | 0.004 | 0.000 | 0.007 | 0.024 |
|  | 2009-2019 | 0.018 | 0.005 | 0.000 | 0.008 | 0.028 |
| 1. **Obstetric haemorrhage** | 1998-2008 | 0.022 | 0.005 | 0.000 | 0.012 | 0.032 |
|  | 2009-2019 | 0.020 | 0.005 | 0.000 | 0.011 | 0.030 |
| 1. **Pregnancy-related infection** | 1998-2008 | 0.009 | 0.003 | 0.008 | 0.002 | 0.015 |
|  | 2009-2019 | 0.009 | 0.004 | 0.009 | 0.002 | 0.016 |
| 1. **Other direct obstetric** | 1998-2008 | 0.016 | 0.005 | 0.001 | 0.007 | 0.025 |
|  | 2009-2019 | 0.013 | 0.004 | 0.002 | 0.004 | 0.021 |
| **7a. HIV & TB** | 1998-2008 | 0.499 | 0.019 | 0.000 | 0.461 | 0.536 |
|  | 2009-2019 | 0.476 | 0.023 | 0.000 | 0.430 | 0.522 |
| **7b. Other infectious diseases** | 1998-2008 | 0.149 | 0.014 | 0.000 | 0.121 | 0.176 |
|  | 2009-2019 | 0.125 | 0.015 | 0.000 | 0.096 | 0.154 |
| **7c. Cardiovascular diseases.** | 1998-2008 | 0.051 | 0.008 | 0.000 | 0.035 | 0.067 |
|  | 2009-2019 | 0.070 | 0.011 | 0.000 | 0.048 | 0.092 |
| **7d. Other NCDs** | 1998-2008 | 0.143 | 0.014 | 0.000 | 0.116 | 0.170 |
|  | 2009-2019 | 0.169 | 0.018 | 0.000 | 0.133 | 0.205 |
| **8. Undetermined** | 1998-2008 | 0.052 | 0.009 | 0.000 | 0.035 | 0.069 |
|  | 2009-2019 | 0.041 | 0.009 | 0.000 | 0.023 | 0.059 |
| **9. Accidents & violence** | 1998-2008 | 0.036 | 0.007 | 0.000 | 0.022 | 0.050 |
|  | 2009-2019 | 0.052 | 0.011 | 0.000 | 0.031 | 0.073 |
| **^a^** There were no deaths in the data attributed to category 6. Unanticipated complications of management, so not shown.  **^b^**  Standard error | | | | | | |

**Figure S6 Multinomial regression predicted proportions for deaths after 42 days postpartum by decade, InterVA5**

**
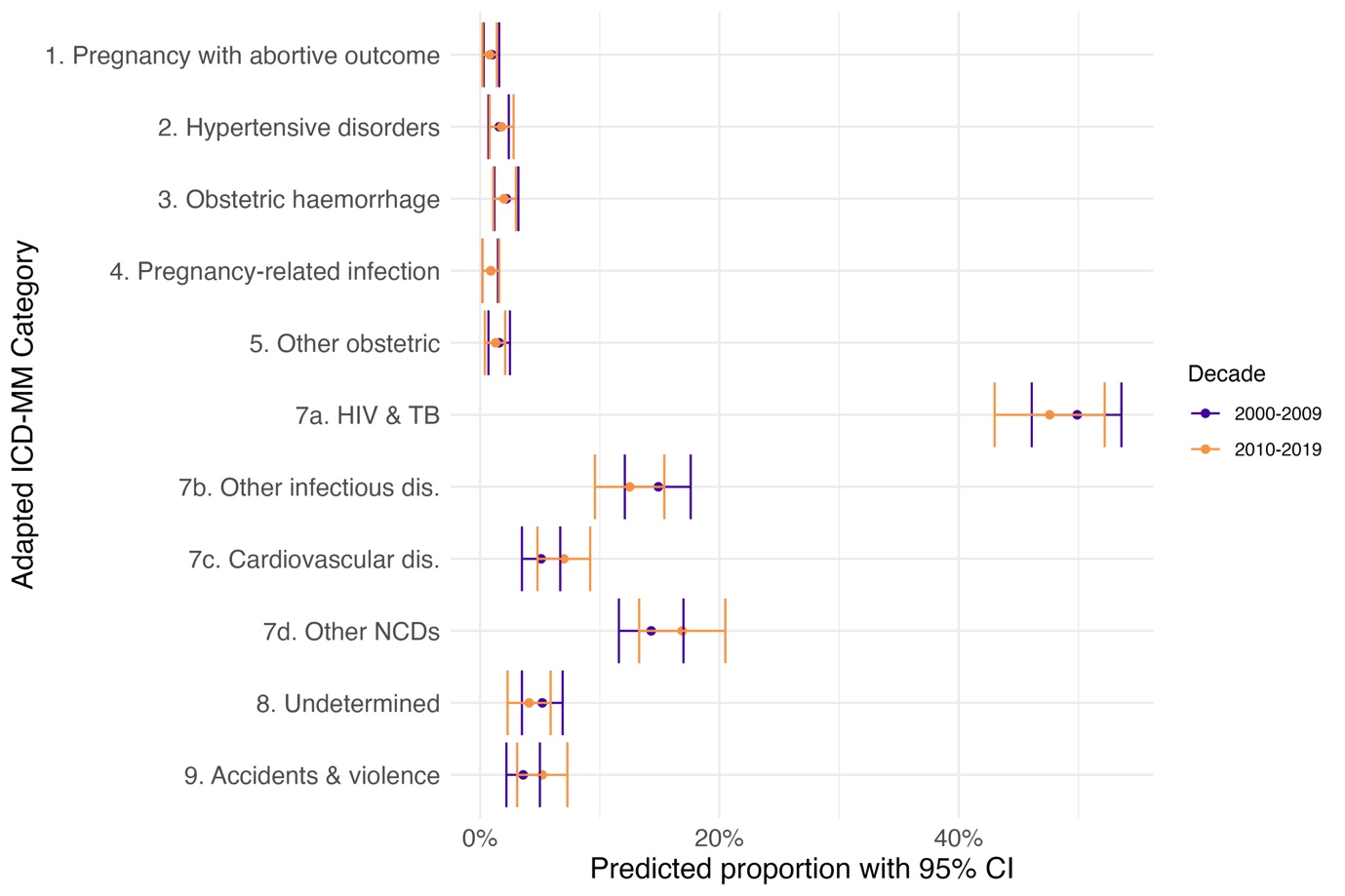
**

**Note:** InterVA attributed no deaths to category 6. Unanticipated complications of management, so not shown.

## Health and Demographic Surveillance Systems

Table S5 and Figure S7 show the predicted proportions from multinomial regression for InterVA for each HDSS. These margins were not stratified by the timing of the death, within or beyond 42 days postpartum, because splitting the data across ten HDSS and two timing categories results in too few deaths in each combination. After accounting for timing and decade, there remain significant differences between at least two HDSS in the predicted proportions of the 11-cause categories for deaths from pregnancy with abortive outcome, hypertensive disorders, obstetric haemorrhage, other direct obstetric, HIV and tuberculosis, other infectious diseases, cardiovascular diseases, and accidental deaths.

For example, the predicted probability of deaths from obstetric haemorrhage is significantly higher in Karonga, Malawi, than in Basse, The Gambia; the predicted proportion of deaths from hypertensive disorders are significantly higher in Basse, The Gambia, than in Kisumu, Kenya or Agincourt, South Africa; the predicted proportions of deaths from HIV and tuberculosis is much greater, and deaths from other infectious diseases much lower, in uMkhanyakude, South Africa, than in other HDSS; and finally, the predicted proportion of deaths from cardiovascular disease is significantly higher in Nairobi, Kenya, than in uMkhanyakude, South Africa.

**Table S5 Predicted margins results by ICD-MM category and HDSS, InterVA5**

| **HDSS** | **Adapted ICD-MM Category^a^** | **Margin** | **SE^b^** | **p-value** | **Lower CI** | **Upper CI** |
| --- | --- | --- | --- | --- | --- | --- |
| **Agincourt, South Africa** | 1. Pregnancy with abortive outcome | 0.004 | 0.004 | 0.316 | -0.004 | 0.013 |
|  | 1. Hypertensive disorders | 0.026 | 0.011 | 0.013 | 0.006 | 0.047 |
|  | 1. Obstetric haemorrhage | 0.231 | 0.025 | 0.000 | 0.182 | 0.280 |
|  | 1. Pregnancy-related infection | 0.004 | 0.004 | 0.316 | -0.004 | 0.013 |
|  | 1. Other direct obstetric | 0.021 | 0.009 | 0.024 | 0.003 | 0.039 |
|  | 7a. HIV & TB | 0.336 | 0.027 | 0.000 | 0.282 | 0.389 |
|  | 7b. Other infectious diseases | 0.086 | 0.017 | 0.000 | 0.052 | 0.119 |
|  | 7c. Cardiovascular diseases. | 0.073 | 0.016 | 0.000 | 0.040 | 0.105 |
|  | 7d. Other NCDs | 0.120 | 0.020 | 0.000 | 0.082 | 0.159 |
|  | 8. Undetermined | 0.069 | 0.016 | 0.000 | 0.038 | 0.100 |
|  | 9. Accidents & violence | 0.030 | 0.010 | 0.004 | 0.009 | 0.050 |
| **Basse,**  **The Gambia** | 1. Pregnancy with abortive outcome | 0.030 | 0.011 | 0.007 | 0.008 | 0.052 |
|  | 1. Hypertensive disorders | 0.108 | 0.020 | 0.000 | 0.069 | 0.147 |
|  | 1. Obstetric haemorrhage | 0.102 | 0.019 | 0.000 | 0.065 | 0.139 |
|  | 1. Pregnancy-related infection | 0.017 | 0.008 | 0.044 | 0.000 | 0.033 |
|  | 1. Other direct obstetric | 0.067 | 0.017 | 0.000 | 0.034 | 0.100 |
|  | 7a. HIV & TB | 0.178 | 0.028 | 0.000 | 0.124 | 0.233 |
|  | 7b. Other infectious diseases | 0.265 | 0.032 | 0.000 | 0.202 | 0.327 |
|  | 7c. Cardiovascular diseases. | 0.072 | 0.017 | 0.000 | 0.038 | 0.106 |
|  | 7d. Other NCDs | 0.137 | 0.025 | 0.000 | 0.088 | 0.185 |
|  | 8. Undetermined | 0.015 | 0.009 | 0.082 | -0.002 | 0.033 |
|  | 9. Accidents & violence | 0.010 | 0.007 | 0.156 | -0.004 | 0.023 |
| **Farafenni, The Gambia** | 1. Pregnancy with abortive outcome | 0.075 | 0.024 | 0.002 | 0.028 | 0.122 |
|  | 1. Hypertensive disorders | 0.078 | 0.025 | 0.002 | 0.029 | 0.126 |
|  | 1. Obstetric haemorrhage | 0.150 | 0.030 | 0.000 | 0.091 | 0.209 |
|  | 1. Pregnancy-related infection | 0.025 | 0.014 | 0.079 | -0.003 | 0.054 |
|  | 1. Other direct obstetric | 0.018 | 0.013 | 0.154 | -0.007 | 0.043 |
|  | 7a. HIV & TB | 0.231 | 0.049 | 0.000 | 0.136 | 0.326 |
|  | 7b. Other infectious diseases | 0.122 | 0.038 | 0.001 | 0.048 | 0.196 |
|  | 7c. Cardiovascular diseases. | 0.075 | 0.028 | 0.006 | 0.021 | 0.129 |
|  | 7d. Other NCDs | 0.162 | 0.043 | 0.000 | 0.077 | 0.247 |
|  | 8. Undetermined | 0.036 | 0.020 | 0.080 | -0.004 | 0.076 |
|  | 9. Accidents & violence | 0.027 | 0.019 | 0.153 | -0.010 | 0.065 |
| **Karonga, Malawi** | 1. Pregnancy with abortive outcome | 0.052 | 0.023 | 0.021 | 0.008 | 0.097 |
|  | 1. Hypertensive disorders | 0.042 | 0.021 | 0.041 | 0.002 | 0.083 |
|  | 1. Obstetric haemorrhage | 0.322 | 0.041 | 0.000 | 0.242 | 0.402 |
|  | 1. Pregnancy-related infection | 0.073 | 0.027 | 0.006 | 0.021 | 0.126 |
|  | 1. Other direct obstetric | 0.033 | 0.019 | 0.078 | -0.004 | 0.069 |
|  | 7a. HIV & TB | 0.204 | 0.041 | 0.000 | 0.123 | 0.286 |
|  | 7b. Other infectious diseases | 0.099 | 0.032 | 0.002 | 0.036 | 0.162 |
|  | 7c. Cardiovascular diseases. | 0.047 | 0.023 | 0.040 | 0.002 | 0.092 |
|  | 7d. Other NCDs | 0.103 | 0.033 | 0.002 | 0.038 | 0.168 |
|  | 8. Undetermined | 0.024 | 0.017 | 0.151 | -0.009 | 0.057 |
|  | 9. Accidents & violence | 0.000 | 0.000 | 0.999 | 0.000 | 0.000 |
| **Kersa, Ethiopia** | 1. Pregnancy with abortive outcome | 0.000 | 0.000 | 0.999 | 0.000 | 0.000 |
|  | 1. Hypertensive disorders | 0.031 | 0.022 | 0.153 | -0.011 | 0.074 |
|  | 1. Obstetric haemorrhage | 0.217 | 0.049 | 0.000 | 0.121 | 0.312 |
|  | 1. Pregnancy-related infection | 0.127 | 0.043 | 0.003 | 0.043 | 0.212 |
|  | 1. Other direct obstetric | 0.037 | 0.026 | 0.155 | -0.014 | 0.088 |
|  | 7a. HIV & TB | 0.185 | 0.050 | 0.000 | 0.087 | 0.284 |
|  | 7b. Other infectious diseases | 0.137 | 0.047 | 0.004 | 0.044 | 0.230 |
|  | 7c. Cardiovascular diseases. | 0.135 | 0.043 | 0.002 | 0.050 | 0.220 |
|  | 7d. Other NCDs | 0.116 | 0.041 | 0.005 | 0.035 | 0.197 |
|  | 8. Undetermined | 0.000 | 0.000 | 0.999 | 0.000 | 0.000 |
|  | 9. Accidents & violence | 0.015 | 0.015 | 0.317 | -0.014 | 0.044 |
| **Kilifi, Kenya** | 1. Pregnancy with abortive outcome | 0.030 | 0.011 | 0.010 | 0.007 | 0.052 |
|  | 1. Hypertensive disorders | 0.076 | 0.017 | 0.000 | 0.042 | 0.110 |
|  | 1. Obstetric haemorrhage | 0.388 | 0.026 | 0.000 | 0.336 | 0.439 |
|  | 1. Pregnancy-related infection | 0.031 | 0.011 | 0.006 | 0.009 | 0.053 |
|  | 1. Other direct obstetric | 0.023 | 0.011 | 0.029 | 0.002 | 0.044 |
|  | 7a. HIV & TB | 0.181 | 0.027 | 0.000 | 0.129 | 0.234 |
|  | 7b. Other infectious diseases | 0.081 | 0.020 | 0.000 | 0.040 | 0.121 |
|  | 7c. Cardiovascular diseases. | 0.050 | 0.015 | 0.001 | 0.022 | 0.079 |
|  | 7d. Other NCDs | 0.077 | 0.019 | 0.000 | 0.040 | 0.114 |
|  | 8. Undetermined | 0.022 | 0.011 | 0.046 | 0.000 | 0.044 |
|  | 9. Accidents & violence | 0.041 | 0.014 | 0.003 | 0.014 | 0.069 |
| **Kisumu, Kenya** | 1. Pregnancy with abortive outcome | 0.023 | 0.006 | 0.000 | 0.011 | 0.036 |
|  | 1. Hypertensive disorders | 0.033 | 0.008 | 0.000 | 0.019 | 0.048 |
|  | 1. Obstetric haemorrhage | 0.271 | 0.017 | 0.000 | 0.238 | 0.303 |
|  | 1. Pregnancy-related infection | 0.031 | 0.007 | 0.000 | 0.017 | 0.046 |
|  | 1. Other direct obstetric | 0.026 | 0.007 | 0.000 | 0.013 | 0.039 |
|  | 7a. HIV & TB | 0.251 | 0.016 | 0.000 | 0.219 | 0.283 |
|  | 7b. Other infectious diseases | 0.115 | 0.013 | 0.000 | 0.091 | 0.140 |
|  | 7c. Cardiovascular diseases. | 0.066 | 0.010 | 0.000 | 0.045 | 0.087 |
|  | 7d. Other NCDs | 0.097 | 0.012 | 0.000 | 0.074 | 0.121 |
|  | 8. Undetermined | 0.055 | 0.009 | 0.000 | 0.037 | 0.073 |
|  | 9. Accidents & violence | 0.030 | 0.007 | 0.000 | 0.016 | 0.044 |
| **Magu, Tanzania** | 1. Pregnancy with abortive outcome | 0.055 | 0.022 | 0.012 | 0.012 | 0.098 |
|  | 1. Hypertensive disorders | 0.046 | 0.020 | 0.022 | 0.006 | 0.085 |
|  | 1. Obstetric haemorrhage | 0.319 | 0.038 | 0.000 | 0.245 | 0.393 |
|  | 1. Pregnancy-related infection | 0.072 | 0.025 | 0.003 | 0.024 | 0.121 |
|  | 1. Other direct obstetric | 0.030 | 0.017 | 0.080 | -0.004 | 0.065 |
|  | 7a. HIV & TB | 0.180 | 0.043 | 0.000 | 0.096 | 0.263 |
|  | 7b. Other infectious diseases | 0.042 | 0.024 | 0.073 | -0.004 | 0.089 |
|  | 7c. Cardiovascular diseases. | 0.034 | 0.019 | 0.079 | -0.004 | 0.071 |
|  | 7d. Other NCDs | 0.142 | 0.039 | 0.000 | 0.065 | 0.219 |
|  | 8. Undetermined | 0.026 | 0.018 | 0.151 | -0.010 | 0.062 |
|  | 9. Accidents & violence | 0.053 | 0.026 | 0.038 | 0.003 | 0.103 |
| **Nairobi, Kenya** | 1. Pregnancy with abortive outcome | 0.093 | 0.023 | 0.000 | 0.048 | 0.139 |
|  | 1. Hypertensive disorders | 0.033 | 0.014 | 0.022 | 0.005 | 0.061 |
|  | 1. Obstetric haemorrhage | 0.144 | 0.027 | 0.000 | 0.092 | 0.197 |
|  | 1. Pregnancy-related infection | 0.007 | 0.007 | 0.315 | -0.006 | 0.019 |
|  | 1. Other direct obstetric | 0.027 | 0.013 | 0.042 | 0.001 | 0.053 |
|  | 7a. HIV & TB | 0.265 | 0.035 | 0.000 | 0.196 | 0.334 |
|  | 7b. Other infectious diseases | 0.107 | 0.026 | 0.000 | 0.056 | 0.157 |
|  | 7c. Cardiovascular diseases. | 0.149 | 0.029 | 0.000 | 0.092 | 0.206 |
|  | 7d. Other NCDs | 0.092 | 0.024 | 0.000 | 0.045 | 0.139 |
|  | 8. Undetermined | 0.042 | 0.017 | 0.012 | 0.009 | 0.075 |
|  | 9. Accidents & violence | 0.042 | 0.017 | 0.012 | 0.009 | 0.074 |
| **uMkhanyakunde,**  **South Africa** | 1. Pregnancy with abortive outcome | 0.032 | 0.011 | 0.004 | 0.010 | 0.054 |
|  | 1. Hypertensive disorders | 0.062 | 0.015 | 0.000 | 0.032 | 0.093 |
|  | 1. Obstetric haemorrhage | 0.113 | 0.020 | 0.000 | 0.074 | 0.152 |
|  | 1. Pregnancy-related infection | 0.013 | 0.007 | 0.082 | -0.002 | 0.027 |
|  | 1. Other direct obstetric | 0.022 | 0.009 | 0.014 | 0.005 | 0.040 |
|  | 7a. HIV & TB | 0.539 | 0.027 | 0.000 | 0.486 | 0.592 |
|  | 7b. Other infectious diseases | 0.025 | 0.008 | 0.003 | 0.009 | 0.041 |
|  | 7c. Cardiovascular diseases. | 0.047 | 0.013 | 0.000 | 0.022 | 0.072 |
|  | 7d. Other NCDs | 0.087 | 0.015 | 0.000 | 0.057 | 0.117 |
|  | 8. Undetermined | 0.021 | 0.008 | 0.008 | 0.005 | 0.036 |
|  | 9. Accidents & violence | 0.040 | 0.011 | 0.000 | 0.018 | 0.062 |
| ^a^ InterVA5 attributed no deaths to category 6. Unanticipated complications of management, so not shown. | | | | | | |

**
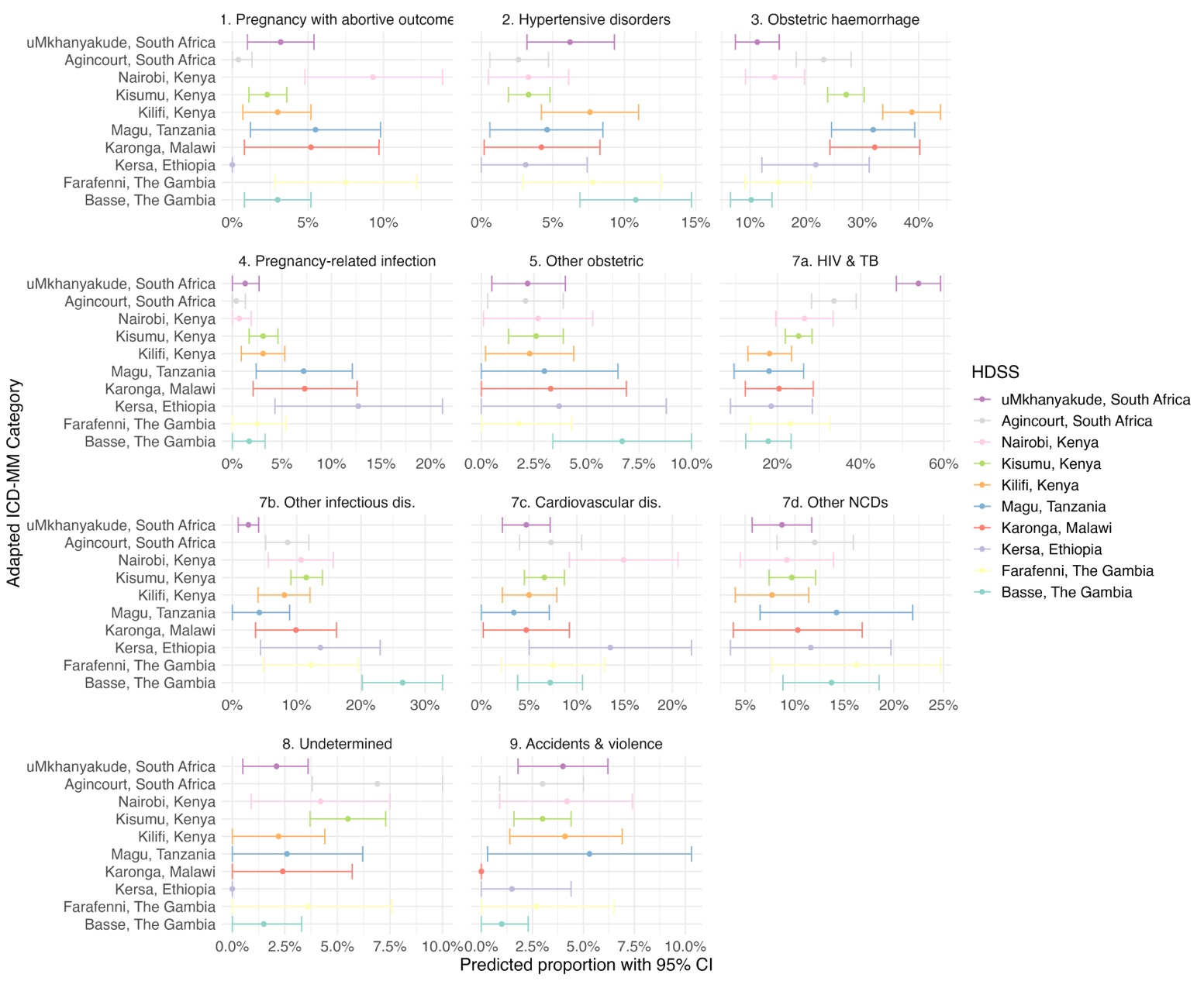
Figure S7 Multinomial regression predicted proportions for deaths after 42 days postpartum by HDSS, InterVA5**

**Note:** InterVA5 attributed no deaths to category 6. Unanticipated complications of management, so not shown.

# Circumstances of Mortality Categories (COMCATs)

The circumstances of deaths within 42 days differ from those which occurred from 43-365 days postpartum. Figure S8 shows that for deaths occurring after (vs. within) 42 days postpartum, fewer were emergencies and more related to either problems receiving care in health systems, or to knowledge, recognition, or awareness of serious disease.

**Figure S8 COMCATs by time of death, InterVA5**


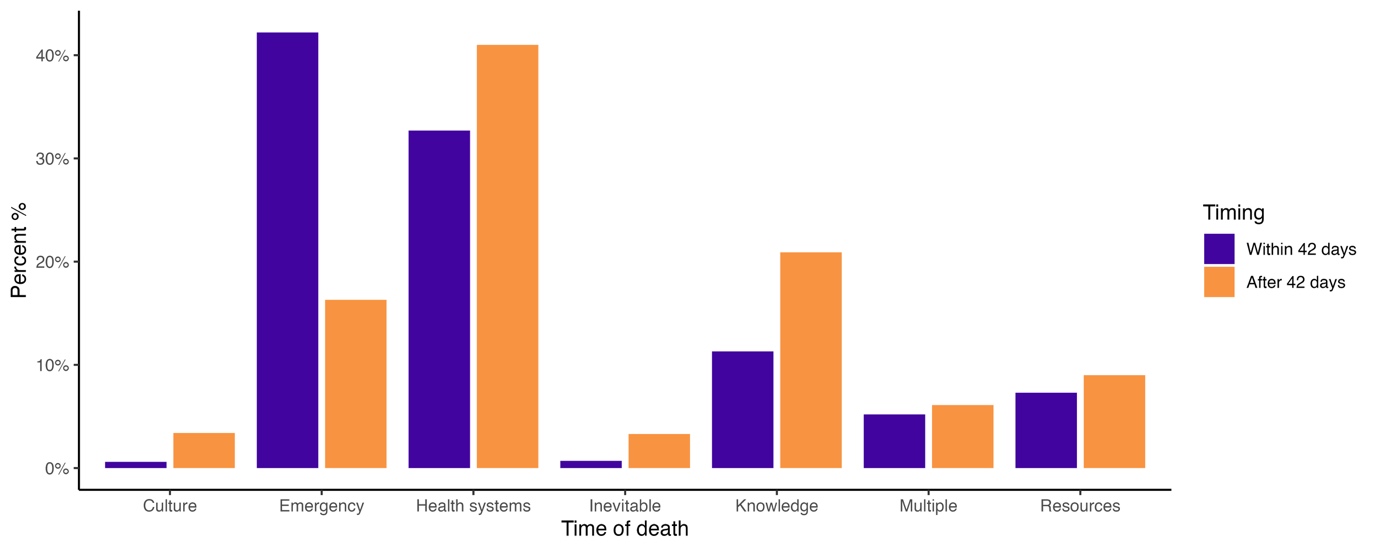


# F . Verbal autopsy coverage by HDSS

#

Coverage of verbal autopsy interviews is not complete for all deaths in all HDSS (Table S6). We calculated the coverage of VA data as the proportion of all deaths, regardless of age and sex, in the HDSS where there was an available VA record. Residency records of deaths in the HDSS and verbal autopsy records were restricted to cover the same time interval.

Coverage estimates are NA for Kilifi and Kersa HDSS because we only had verbal autopsy data for deaths to pregnant or recently pregnant women in those two sites.

**Table S6 Coverage of VA data by HDSS**

| **HDSS** | **Coverage (%)** | **Years of deaths in the sample** |
| --- | --- | --- |
| Agincourt, South Africa | 84 | 2000-2017 |
| Basse, The Gambia | 33 | 2006-2019 |
| Farafenni, The Gambia | 26 | 2000-2019 |
| Kersa, Ethiopia | NA | NA |
| Kilifi, Kenya | NA | NA |
| Kisumu, Kenya | 57 | 2003-2013 |
| Magu, Tanzania | 42 | 2000-2017 |
| Nairobi, Kenya | 100 | 2002-2016 |
| Karonga, Malawi | 100 | 2002-2017 |
| uMkhanyakude, South Africa | 100 | 2000-2017 |

In all eight HDSS with available data, some verbal autopsy records have no corresponding match in a death record. In Nairobi, Karonga, and uMkhanyakude, this means coverage exceeds 100%. This may occur for two reasons:

1. Some verbal autopsy interviews are conducted for non-residents of the site
2. Changes to the individual unique ID numbers prevents record merges.

Incompleteness in Agincourt, Basse, Farafenni, Kisesa, Kisumu and Magu may occur if:

1. Not all deaths in the HDSS are followed-up with a verbal autopsy interview
2. Changes in individual unique ID numbers prevent merges of death and VA records

Figures S9 shows the changes in coverage of verbal autopsy records for deaths in the HDSS by year. Coverage varies significantly over time in all three HDSS. In the HDSS in The Gambia, coverage has steadily declined – in Basse from 2010, and in Farafenni from 2003. In Agincourt and Magu, coverage has increased over time.

**Figure S9 Verbal autopsy coverage for deaths by year**


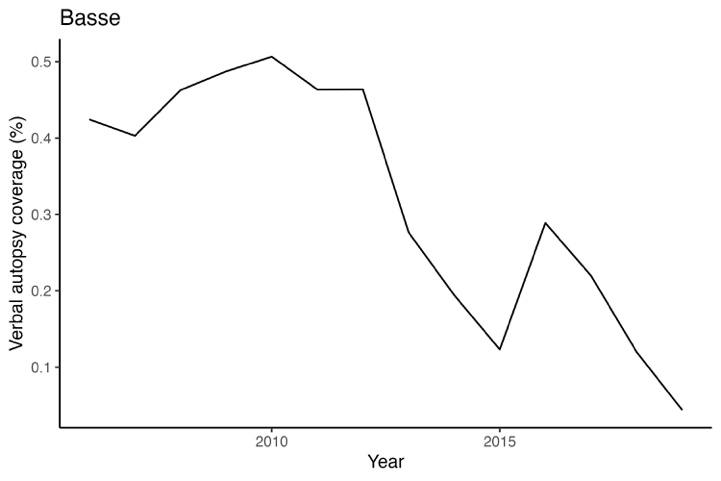

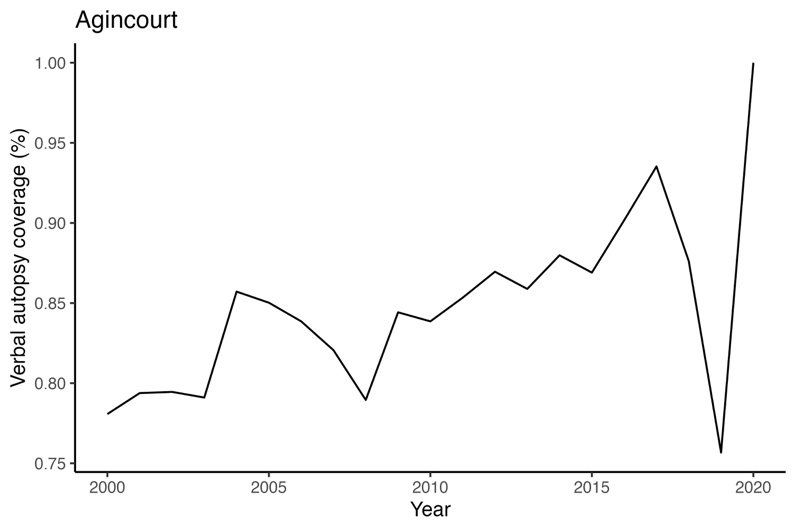


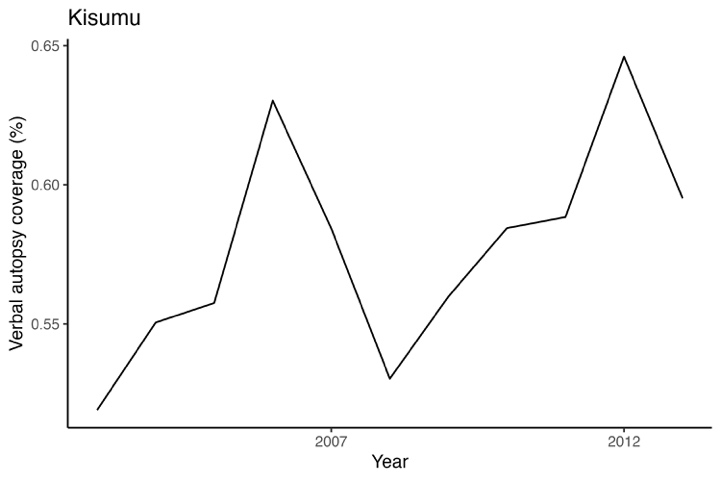

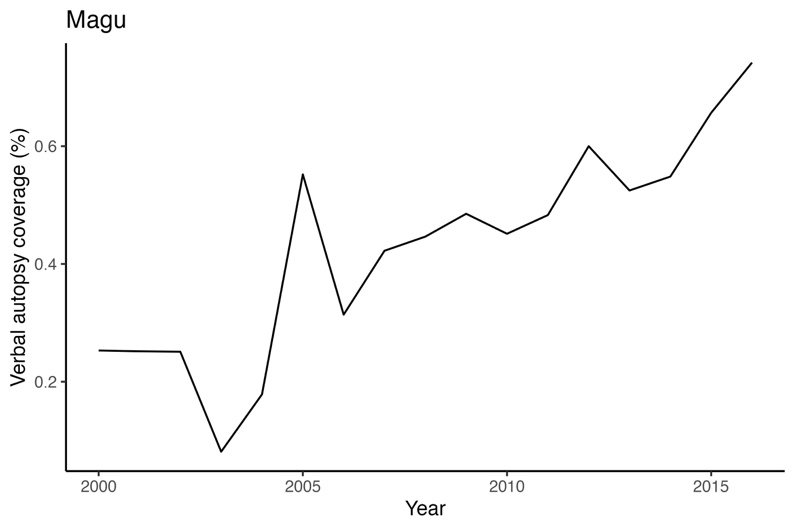

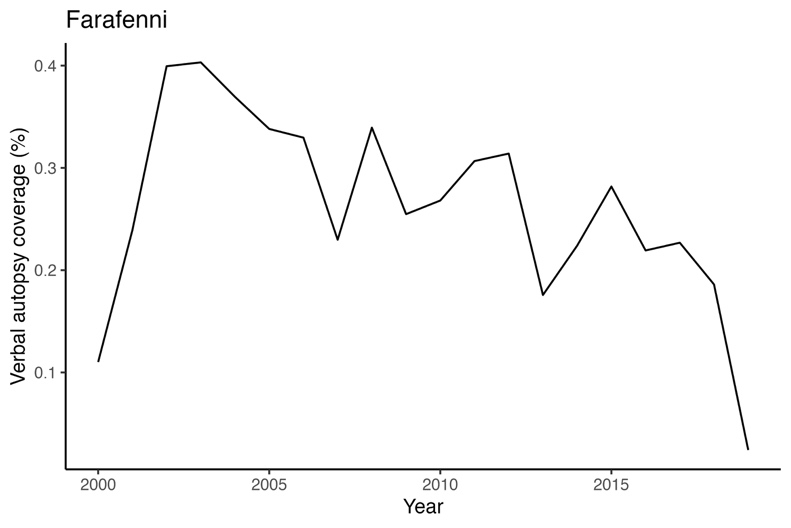

Supplement: Supplementary file 1 — Appendix S1. [file BJO-131-163-s001.docx]
